# Extracellular Vesicle‐Encapsulated Small RNAs From *Clostridium Butyricum* Modulate Host Intestinal Lipid Metabolism

**DOI:** 10.1002/jev2.70381

**Published:** 2026-09-28

**Authors:** Hong Hu, Yiwen He, Jing Liang, Qi Han, Xuqing Liang, Guangtian Cao, Yulong Tang, Liuqin He, Yulong Yin, Xihong Zhou

**Affiliations:** ^1^ Institute of Subtropical Agriculture the Chinese Academy of Sciences Changsha China; ^2^ College of Animal Science and Technology Hunan Agricultural University Changsha China; ^3^ Hunan Provincial Key Laboratory of Animal Intestinal Function and Regulation College of Life Sciences Hunan Normal University Changsha China; ^4^ College of Advanced Agricultural Sciences University of Chinese Academy of Sciences Beijing China; ^5^ College of Bioscience and Biotechnology Hunan Agricultural University Changsha China; ^6^ College of Standardisation China Jiliang University Hangzhou China; ^7^ Yuelushan Laboratory Changsha China

**Keywords:** *Clostridium butyricum*, Extracellular vesicles, Gut microbiota, Lipid metabolism, Obesity, Organoids

## Abstract

The gut microbiota plays a critical role in regulating intestinal lipid metabolism, yet the mechanisms governing microbiota–host interactions during lipid uptake remain unclear. Here, we examined the relationship between fat digestibility and gut microbial composition, and found that piglets with higher apparent crude fat digestibility exhibited enhanced lipid absorption and oxidation, accompanied by reduced abundance of *Clostridium butyricum* in the ileal mucosa. Furthermore, oral administration of *C. butyricum* suppressed intestinal lipid absorption and oxidation by inhibiting PPARα signaling. Mechanistically, extracellular vesicles (EVs) derived from *C. butyricum* can enter intestinal epithelial cells and mediate the anti‐lipid absorption effects by delivering miRNA‐like RNAs (milRNAs), particularly milRNA‐3020179, which primarily targeted host genes including *PPARα* and *Rxra*. Supplementation with *C. butyricum* or its EVs prevented lipid accumulation and attenuated the development of metabolic abnormalities in high‐fat diet‐fed mice, whereas deletion of milRNA‐3020179 in *C. butyricum* abolished its effects on lipid deposition in intestinal organoids. Collectively, these findings identify *C. butyricum*‐derived EV milRNAs as candidate microbial regulators of host lipid metabolism through modulation of PPARα signaling, providing insights into microbiota‐driven control of intestinal lipid homeostasis.

## Graphical abstract

Extracellular vesicles (EVs) derived from *C. butyricum* can enter intestinal epithelial cells and mediate the anti‐lipid absorption effects by delivering miRNA‐like RNAs (milRNAs), particularly milRNA‐3020179, which primarily targeted host genes majorly including PPARα. Deletion of milRNA‐3020179 in *C. butyricum* abolished its effects on lipid deposition in intestinal organoids.

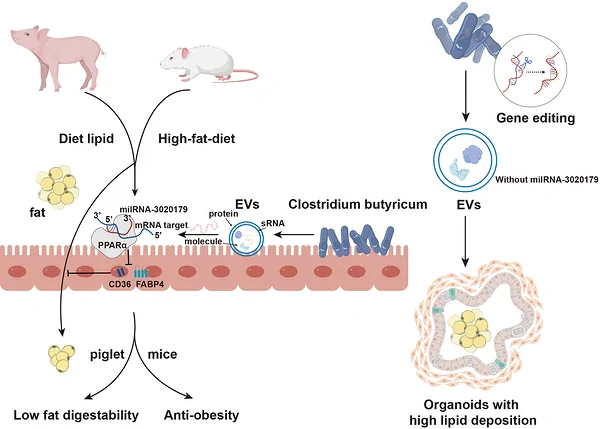

## 1 Introduction

The small intestine is the primary site for dietary lipid absorption, and intestinal lipid metabolism is closely linked to diet‐induced obesity and metabolic syndromes. Therefore, molecular regulators of intestinal lipid metabolism represent important therapeutic targets for these conditions (Dávalos‐Salas et al. 2020, Ko et al. 2020). For instance, Cluster of Differentiation 36 (CD36) and fatty acid transport protein 4 (FATP4), which function as key lipid sensors and transporters, play critical roles in lipid absorption and the development of obesity (Lebrun et al. 2022). In addition, the peroxisome proliferator‐activated receptor (PPAR) family of nuclear receptors serves as central regulators of fatty acid oxidation and lipid uptake (Dávalos‐Salas et al. 2020). Notably, several factors have been reported to protect against obesity by modulating intestinal lipid metabolism. For instance, deletion of a nuclear receptor corepressor in intestinal epithelial cells ameliorates obesity by impairing duodenal lipid absorption (Hou et al. 2024). Similarly, the long noncoding RNA small nucleolar RNA host gene 9 suppresses PPARγ, leading to impaired lipid absorption and protection against diet‐induced obesity (Wang et al. 2023). In addition, phenyllactic acid, a metabolite produced by *Lactobacillus*, regulates intestinal PPARγ to limit fat accumulation and confer protection against obesity (Shelton et al. 2023). Collectively, these findings highlight intestinal lipid metabolism as a promising therapeutic target for the treatment of obesity and related metabolic syndromes.

Gut microbiota, particularly that of the small intestine, is also a critical determinant of lipid absorption and metabolism and is closely linked to obesity development. Germ‐free mice fed a high‐fat diet (HFD) exhibit elevated fecal lipid content and show resistance to obesity (Rabot et al. 2010), whereas colonization of germ‐free mice with intestinal microbiota from obese donors increases total body fat in the recipients despite unchanged dietary intake (Kallus and Brandt 2012). These observations suggest that the absence of microbiota leads to reduced intestinal fat digestion and absorption, coupled with enhanced fat oxidation. Moreover, specific small intestinal microbial taxa, such as *Lactobacillaceae* and *Clostridiaceae*, have been shown to inhibit lipid absorption and secretion, thereby limiting serum triglyceride (TG) levels in response to HF diets (Shelton et al. 2023, Martinez‐Guryn et al. 2018). Although several studies have demonstrated that gut microbes can modulate host genes involved in lipid metabolism through their metabolites (Shelton et al. 2023, Martinez‐Guryn et al. 2018, Lin and Wang 2017), emerging evidence suggests that microbe‐derived extracellular vesicles (EVs) also serve as critical mediators of host–microbe interactions. In particular, EVs carry small RNAs, including microRNA‐like RNAs (milRNAs), which are similar in size to eukaryotic miRNAs, possess stem–loop structures, and regulate gene expression by targeting mRNAs (Cardin and Borchert 2017). MilRNAs have been identified in fungi, viruses, and bacteria (Cardin and Borchert 2017, Zhou et al. 2012, Yu et al. 2022); however, their role in regulating host intestinal lipid metabolism remains largely unknown.

Therefore, we employed weanling piglets as a physiologically appropriate and well‐established model, characterized by active lipid metabolic development (Li et al. 2024), ongoing gut microbiota maturation (Gresse et al. 2017), and dynamic host–microbiota interactions in nutrient absorption (St‐Pierre et al. 2023). Initial evaluation of fat apparent digestibility and gut microbiota composition revealed that colonization of *Clostridium butyricum* in the proximal ileal mucosa was enriched in individuals with lower fat digestibility. Further investigation revealed that *C. butyricum* suppresses intestinal lipid absorption and oxidation by downregulating PPARα signaling. Notably, EVs derived from *C. butyricum* play a central role in modulating intestinal lipid metabolism. Analysis of the RNA content within these EVs identified three milRNAs that potentially target genes involved in lipid metabolism in HFD‐fed mice. Finally, using organoid models, we confirmed that milRNA‐3020179 carried by EVs is the candidate regulator of intestinal fat absorption, as deletion of its coding sequence in *C. butyricum* abolished this effect. Collectively, these findings demonstrate that EV‐encapsulated small RNAs from *C. butyricum* can modulate host intestinal lipid metabolism, with potential implications for conditions of over‐nutrition.

## 2 Materials and Methods

### 2.1 Ethics Statement

Animal procedures were approved by the Protocol Management and Review Committee of the Institute of Subtropical Agriculture, Chinese Academy of Sciences. All animal experiments were approved under the general ethical approval number 20230126. The specific ethical approval numbers for individual animal experiments were 20230126‐1, 20230126‐2, 20230126‐3, 20230126‐4, 20230126‐5, 20230126‐6, and 20230126–7, respectively.

### 2.2 Animals

Crossbred piglets (Duroc × Landrace × Yorkshire), weaned at 21 days of age, were obtained from Hunan New Wellful Co., Ltd. (Changsha, China). Throughout the experiment, all piglets had free access to feed and water and were provided diets formulated according to the Nutrient Requirements of Swine (2012). At the end of the trial, piglets were humanely anesthetized with sodium pentobarbital (50 mg/kg body weight, intravenous injection) and euthanized by exsanguination to minimize suffering (Ethical approval No. 20230126‐1). In addition, seven‐week‐old male C57BL/6 mice were supplied by Slac Laboratory Animal Central (Changsha, China) and housed under specific pathogen‐free (SPF) conditions.

### 2.3 Determination of Crude Fat Apparent Digestibility

A dose of 0.4% titanium dioxide (TiO_2_) was incorporated into the diet as an indigestible marker to assess apparent fat digestibility. One hundred piglets weaned at 21 days of age were used for fecal collection from day 22 to 24. Feces from each piglet were homogenized, dried at 65°C for 72 h, and analyzed for crude fat content using an automated extraction analyzer (XT 15i, Ankom Technology, USA). The concentration of TiO_2_ was determined as previously described (Ma et al. 2023). The crude fat apparent digestibility was calculated as follow: Apparent nutrient digestibility (%) = [1 – (TiO2 content in the diet/TiO2 content in the feces) × (fat content in the feces/fat content in the diet)] × 100%.

### 2.4 Determination of TG, Total Cholesterol (TC) and Non‐esterified Fatty Acids (NEFA) Content

TG, TC and NEFA were measured using commercial kits (BioSino, Beijing, China; Sigma, Shanghai, China). For tissue lipid analysis, approximately 20 mg of tissue was homogenized in 5% Triton X‐100 saline. For fecal lipid analysis, 0.5 g of feces was dried at 60°C for 48 h, weighed, and ground. The samples were then extracted with methanol: chloroform (1:2, v/v), shaken for 12 h, and subsequently dissolved in isopropanol containing 10% Triton X‐100 saline. TG, TC, and NEFA concentrations were finally determined using the aforementioned kits.

### 2.5 Determination of Total Bile Acid, Secretin (SCT) and Cholecystokinin (CCK) Content and Lipase Activity

For the determination of serum bile acid content, 50 µL of serum was used. For intestinal bile acid analysis, 20 mg of jejunal or ileal contents were weighed and homogenized in 100 µL of ddH_2_O. Total bile acid concentrations were then measured using commercial kits (Mlbio, Shanghai, China). SCT and CCK levels, as well as lipase activity, were assessed using commercial ELISA kits (Boyan, Nanjing, China).

### 2.6 Quantitative Reverse Transcription PCR (RT‐qPCR) Analysis

Collected tissues stored at −80°C were used for total RNA extraction with TRIzol reagent (Invitrogen, Shanghai, China). The extracted RNA was then reverse‐transcribed into cDNA using the High‐Capacity cDNA Reverse Transcription Kit (Applied Biosystems, Shanghai, China). Quantitative PCR was performed in a total reaction volume of 20 µL containing cDNA, primers, and SYBR qPCR Master Mix (Applied Biosystems) on a Roche LightCycler 480 II system (Indianapolis, IN, USA). Relative mRNA expression levels were determined using the comparative Ct method (2^−ΔΔCt^) and normalized to β‐actin. Primer sequences are provided in Table S1.

### 2.7 Microbiota Profiling and C. butyricum Quantification

Genomic DNA was extracted from intestinal contents and mucosa using the Stool DNA Kit (TianGen, Beijing, China) and the CTAB method, respectively. The V3‐V4 region of the 16S rRNA gene was amplified using primers with unique barcodes. PCR reactions were performed with Phusion High‐Fidelity PCR Master Mix (New England Biolabs, Shanghai, China). Sequencing libraries were constructed using the NEB Next Ultra II FS DNA PCR‐free Library Prep Kit (New England Biolabs). Paired‐end reads were assigned to samples according to their unique barcodes using Python (v3.6.13). High‐quality clean tags were obtained by quality filtering of the raw reads using *fastp* (v0.23.1). Sequence analysis was conducted using Uparse (v7.0.1001, http://drive5.com/uparse/), and sequences with ≥97% similarity were clustered into operational taxonomic units (OTUs). OTU abundance data were normalized for downstream alpha‐ and beta‐diversity analyses. The top 10 taxa at each taxonomic rank were selected using Perl with the SVG function. Principal coordinate analysis (PCoA) was performed with the *ade4* and *ggplot2* packages in R (v4.0.3). For quantification of *C. butyricum* abundance, PCR was conducted in a 20 µL reaction system containing TaqMan Universal PCR Master Mix (Applied Biosystems), genomic DNA, and specific primer pairs. Primer sequences are listed in Table S2.

### 2.8 Mucosal Microbiota Transplantation

Jejunum and ileum samples were rinsed twice with sterile PBS to remove residual contents, and the mucosa was collected by scraping the tissue surface with a cell scraper (Greiner, Shanghai, China) into fresh, ice‐cold PBS. The samples were centrifuged at 50 *g* for 15 min at 4°C to remove debris, and the supernatants were filtered through 70 µm strainers into new tubes. A volume of 0.1 mL of the filtered supernatant was orally administered to recipient mice by gavage once daily for 21 consecutive days (Ethical approval No. 20230126‐2). The recipient mice were pretreated with a mixture of antibiotics as previously described (He et al. 2022).

### 2.9 Determination of Fatty Acid Oxidation (FAO)

Mitochondria were isolated from the intestine as previously described (Dong et al. 2024), and FAO was assessed using a fatty acid complete oxidation assay kit (Abcam, Shanghai, China). Briefly, equal amounts of isolated mitochondria were incubated in assay medium containing 0.5 mM L‐carnitine and 150 µM oleate conjugated to BSA. Following the addition of the extracellular oxygen reagent, fatty acid‐driven oxygen consumption was quantified by monitoring the increase in fluorescence at an excitation wavelength of 380 nm and an emission wavelength of 650 nm.

### 2.10 Oral Administration of C. butyricum

Fecal samples from pigs were freshly collected and placed in 5 mL of Reinforced Clostridial Medium (RCM) liquid medium, overlaid with liquid paraffin. After incubation for 48 h at 37°C in an anaerobic chamber, the bacterial suspension was spread onto RCM agar plates and further incubated for 24 h at 37°C. Single colonies were then selected for 16S rRNA sequencing to identify the isolates; one strain was confirmed as *C. butyricum* and the complete genome was subsequently sequenced using the PacBio Sequel and Illumina HiSeq X Ten platforms. Finally, 0.1 mL of *C. butyricum* suspension (10^9^ CFU) was orally administered to mice once daily for two weeks (Ethical approval No. 20230126‐3).

### 2.11 Lipid Absorption Analysis

For the TG tolerance test, mice were fasted overnight and then orally gavaged with olive oil at a dose of 10 µL/g body weight. Blood samples were collected at 0, 1, 2, 3, and 4 h post‐gavage for determination of serum TG levels. For assessment of lipoprotein lipase (LPL)‐mediated inhibition of lipid absorption, animals were intraperitoneally injected with poloxamer 407 (1.0 g/kg body weight; Sigma Aldrich) and orally gavaged with 200 µL olive oil. Blood was collected at 0, 1, 2, 3, and 4 h after gavage for TG measurement.

### 2.12 Transcriptome Analysis

Total RNA was extracted using TRIzol reagent, and RNA quality was assessed with a 5300 Bioanalyzer (Agilent, California, PA, USA). PCR amplification was performed using Phusion High‐Fidelity DNA Polymerase, and the resulting products were purified for library construction. Sequencing libraries were then generated and sequenced on an Illumina NovaSeq platform, producing 150 bp paired‐end reads for downstream analysis. Differentially expressed genes (DEGs) were identified using the DESeq2 R package (v1.20.0). Gene Ontology (GO) enrichment and Kyoto Encyclopedia of Genes and Genomes (KEGG) pathway analyses were performed using Goatools and Python (v3.12), respectively.

### 2.13 Western Blotting Analysis

Total protein was extracted from intestinal tissues and cells, followed by denaturation and separation via SDS‐PAGE. Proteins were then transferred onto nitrocellulose membranes. Membranes were incubated with primary antibodies against PPARα, PPARγ, PPARδ, Retinoid X receptor beta (Rxrb), Fatty acid binding protein 2 (Fabp2), GroEL, GAPDH, β‐actin (Abcam, Shanghai, China), Rxra, Lpl, CD36 (Bioss, Beijing, China), Carnitine palmitoyltransferase 1a (CPT1a) and Fatty acid transport protein 4 (Fatp4) (Boster, Wuhan, China). Subsequently, membranes were incubated with appropriate secondary antibodies, and protein bands were visualized using EZ‐ECL chemiluminescence reagent (Biological Industries, Cromwell, CT, USA).

### 2.14 Sodium Butyrate Treatment

Mice were orally administered either sodium butyrate (200 mg/kg body weight, dissolved in 150 µL saline) or an equivalent volume of saline once daily for two weeks (Ethical approval No. 20230126‐4).

### 2.15 Isolation and Identification of EVs Derived From C. butyricum

*C. butyricum* was cultured in RCM medium for 16 h to reach the logarithmic growth phase. The conditioned medium was then collected and sequentially centrifuged at 1000 *g*, 3000 *g*, and 10,000* g* for 30 min at 4°C. The supernatant was filtered through a 0.22 µm membrane to remove large particles and residual bacteria, followed by ultracentrifugation at 150,000 *g* for 3 h at 4°C (Beckman Coulter 100XP, CA, USA). The pellet was resuspended in 100 µL PBS. The size and concentration of EVs were determined using nanoparticle tracking analysis (NTA) using a ZetaView instrument (Particle Metrix, Munich, Germany) and transmission electron microscopy (TEM) (Hitachi HT‐7700, Tokyo, Japan). Protein content of EV preparations was quantified using a BCA protein assay kit (Beyotime, Shanghai, China). The particle‐to‐protein ratio ranged from 3.7 × 10^9^ to 4.0 × 10^9^ particles per µg protein. For subsequent experiments, EV dosage was normalized based on protein content. EV samples were stored at −80°C until use.

EVs were labeled with either DiI or DiR fluorescent dye and subsequently purified to remove residual free dye and dye micelles, as illustrated in Figure S1. Briefly, purified EVs were incubated with 5 µM DiI (Solarbio, Beijing, China) or DiR (Dojindo, Shanghai, China) at 37°C for 20 min in the dark. Following labeling, the samples were ultra‐centrifuged at 150,000 *g* for 3 h at 4°C to remove unbound dye. The resulting EV pellet was resuspended in 100 µL sterile PBS. To further eliminate residual free dye and dye aggregates, the labeled EVs were subjected to OptiPrep density gradient purification. A discontinuous iodixanol gradient consisting of 50%, 40%, 30%, 20%, and 10% (w/v) OptiPrep solutions was carefully prepared in ultracentrifuge tubes (Beckman Coulter) by layering the solutions sequentially from the bottom to the top. The 100 µL suspension of dye‐labeled EVs was gently loaded onto the top of the gradient and centrifuged at 150,000 *g* for 3 h at 4°C. After centrifugation, the EV‐containing fraction was collected from the interface between the 20% and 30% iodixanol layers, whereas denser non‐vesicular materials and dye aggregates remained in lower‐density fractions, primarily below the 40% iodixanol layer, and were discarded. The collected EV fraction was diluted in 25 mL PBS and ultra‐centrifuged again at 150,000 *g* for 3 h at 4°C to remove iodixanol and any residual free dye. Finally, the purified EV pellet was resuspended in 100 µL PBS and stored at −80°C until further use. To verify the effectiveness of the purification procedure, free DiI dye subjected to the same labeling and purification protocol in the absence of EVs was used as a negative control.

### 2.16 EVs Treatment on Mice

To track the in vivo biodistribution of *C. butyricum*‐derived EVs, EVs were first labeled with the dye DiR (Dojindo). Briefly, 100 µg protein of EVs were incubated with 5 µM DiR in PBS at 22 ± 2°C for 30 min in the dark, followed by removal of ultracentrifugation at 150,000 *g* for 1 h at 4°C. Free DiR dye (5 µM) was prepared as a control in the same buffer without EVs. C57BL/6 mice were fasted for 4 h prior to oral administration. Animals were randomly assigned to two groups: DiR‐labeled EVs (100 µg protein) and free dye (5 µM). Animals were sacrificed at 3, 6, and 24 h post‐administration. The stomach and the whole intestine were carefully excised and placed in petri dishes for imaging. Fluorescent signals were recorded using an in vivo imaging system (FUSION FX7 EDGE SPECTRA, Vilber, Collegien, France). To investigate the effects of *C. butyricum*‐derived EVs on intestinal lipid metabolism, mice were orally administered EVs (100 µg protein) once daily for two consecutive weeks (Ethical approval No. 20230126‐5).

### 2.17 EVs Treatment on Intestinal Epithelial Cells

Firstly, cell viability was assessed using a Cell Counting Kit‐8 (CCK‐8, Dojindo, Japan) according to the manufacturer's instructions. IPEC‐J2 cells were seeded in 96‐well plates at a density of 5 × 10^3^ cells per well and cultured overnight at 37°C in a humidified incubator with 5% CO_2_. Cells were then treated with EVs (dissolved in 10 µL PBS) at the indicated concentrations for 24 h and 48 h. Control cells received an equal volume of PBS. After treatment, 10 µL of CCK‐8 solution was added to each well (a total volume of 100 µL) and incubated for 1–2 h at 37°C. The absorbance at 450 nm was measured using a microplate reader. Cell viability was expressed as a percentage relative to the control group.

To assess the uptake of EVs by IPEC‐J2 cells, EVs were freshly prepared, gently resuspended, centrifuged, and then labeled with DiI dye. Unincorporated fluorescent dye was carefully removed as described in Figure S1 before uptake experiments. DiI‐labeled EVs (50 µg protein) were incubated with the cells for 12 h, and then washed extensively with cold PBS (3 times, 2 mL per well for 6‐well plates) to remove uninternalized or membrane‐bound EVs prior to imaging. Free DiI dye processed identically in the absence of EVs was used to treat cells as a negative control. In addition, free DiI dye (0.5 µM) was directly added to cells as a positive control. Cells were further fixed in 4% paraformaldehyde (Solarbio) for 30 min. After three additional PBS washes, the cells were incubated with anti‐β‐actin (Abcam, Shanghai, China) and DAPI (Solarbio), respectively, and visualized using fluorescence microscopy (LSM880, Zeiss). To investigate the effects of *C. butyricum*‐derived EVs on intestinal epithelial cell lipid metabolism, EVs (50 µg protein) were added to the culture medium (a total volume of 2 mL) for 24 h.

### 2.18 Small RNA Sequencing

EVs isolated from *C. butyricum* were used for RNA extraction and purification with the exoRNeasy Maxi Kit (Qiagen, Shanghai, China). A total of 10 ng RNA per sample was used for library preparation with the Small RNA Library Prep Kit for Illumina (Abclonal, Wuhan, China). cDNA was synthesized using M‐MuLV Reverse Transcriptase, followed by PCR amplification with LongAmp Taq 2 × Master Mix, SR Primer for Illumina, and index (X) primers. Sequence were analyzed by Novogene Co., Ltd. (Beijing, China) and the distribution of sRNAs was analyzed with Bowtie after length screening against the reference sequence of *C. butyricum*. To identify known microRNAs, unique sequences with lengths ranging from 18 to 26 nucleotides were mapped to species precursors in miRBase 22.0, and novel microRNAs were predicted using miRDeep2 and miREvo.

### 2.19 Identification of miRNA by qPCR Analysis

Total RNA extracted from *C. butyricum*‐derived EVs was used for miRNA cDNA synthesis with the TaqMan Advanced miRNA cDNA Synthesis Kit (Applied Biosystems, Shanghai, China). PCR was performed using the TaqMan Advanced miRNA Assay Kit (Applied Biosystems) on a Roche LightCycler 480 II system (Indianapolis, IN, USA), following the manufacturer's instructions. Primer sequences are listed in Table S3. PCR products were analyzed by agarose gel electrophoresis.

### 2.20 RNase A, DNase, and Proteinase K Treatment on EVs

RNase A, DNase, and proteinase K treatment was performed as previously described with minor modifications (Fan et al. 2024, Enderle et al. 2015). For DNase treatment, EVs resuspended in PBS were incubated with DNase I (TIANGEN, Beijing, China) in the presence of 0.3% Triton X‐100 at 22 ± 2°C for 15 min, and then heat‐inactivated at 75°C for 5 min. For RNase A treatment, EVs were incubated with RNase A (Thermo Fisher Scientific, Shanghai, China) with 0.3% Triton X‐100 at 22 ± 2°C for 30 min. RNase A was subsequently inactivated by autoclaving. To control for potential effects of the enzyme solvent, parallel samples were treated with an equivalent volume of RNase A buffer together with Triton X‐100 (control treatment). Additional control groups included EVs treated with RNase A alone, Triton X‐100 alone, or the combination of RNase A and Triton X‐100. RT‐qPCR analysis was then conducted to determine whether milRNAs were protected within EVs and degraded by RNase A upon membrane disruption. For proteinase K treatment, EVs were incubated with proteinase K (TIANGEN, Beijing, China) in the presence of 0.3% Triton X‐100 at 22 ± 2°C for 20 min, followed by heat inactivation at 75°C for 10 min.

### 2.21 Separation of Small RNA Fractions

Small RNA fractions were separated by denaturing polyacrylamide gel electrophoresis. Total RNA was mixed with RNA loading buffer and resolved on a 15% polyacrylamide gel containing 7 M urea in 1× TBE buffer. After electrophoresis, the gel was stained with SYBR Gold, and RNA bands were visualized under UV illumination. Gel regions corresponding to 20–40 nt and 40–200 nt RNA fragments were excised separately, and RNA was recovered using a standard gel extraction procedure for subsequent analyses.

### 2.22 MilRNA Mimics Treatment on Intestinal Epithelial Cells

MilRNA mimics, Cy5‐labeled milRNAs mimics and the corresponding negative controls were synthesized by Sangon Biotech (Shanghai, China). IPEC‐J2 cells were transfected with 50 nM mimics or Cy5‐labeled mimics using Lipofectamine 2000 transfection reagent (Invitrogen), according to the manufacturer's instructions.

### 2.23 MilRNA Agomir Treatment in Mice

MilRNA agomirs were synthesized by Sangon Biotech (Shanghai, China). Mice were intraperitoneally injected with either a mixture of milRNA agomirs targeting milRNA‐409778, milRNA‐3020179, and milRNA‐3047216, or a negative control (NC). All agomirs and NC were dissolved in PBS and administered in a total volume of 100 µL per injection. Sequence information are provided in Table S4. The antisense strand of agomirs and NC were chemically modified as follows: a cholesterol moiety was conjugated to the 3′ end; two phosphorothioate backbone modifications were introduced at the 5′ end; four phosphorothioate backbone modifications were introduced at the 3′ end; and the entire strand was modified with 2′‐O‐methyl groups. Each mouse received a mixture of three milRNA agomirs (milRNA‐409778, milRNA‐3020179, and milRNA‐3047216), with each agomir administered at 20 mg/kg body weight (total dose: 60 mg/kg body weight). Mice in the negative control (NC) group received NC at the same total dose (60 mg/kg body weight), while mice in the control group received an equal volume of PBS. The treatment was performed once daily for 7 consecutive days (Ethical approval No. 20230126‐6).

### 2.24 Treatment of *C. butyricum* and Its Derived EVs on High‐fat Diet‐Fed Mice

All mice were randomly assigned to four groups: a low‐fat diet control group (CON); a high‐fat diet group (HFD); a high‐fat diet group orally administered 0.1 mL of *C. butyricum* suspension (1 × 10^9^ CFU) every two days (CLO); and a high‐fat diet group orally administered 100 µg protein of EVs every two days (EVS). The mice were simultaneously subjected to HFD feeding and administration of *C. butyricum* or EVs from the beginning of the experiment. The selected dose was based on previous studies (Chen et al. 2026, Park et al. 2025) and our preliminary experiments. The experimental period lasted 60 days (Ethical approval No. 20230126–7). The low‐fat diet provided 10% of total energy from fat, 20% from protein, and 70% from carbohydrates, whereas the high‐fat diet provided 45% of total energy from fat, 20% from protein, and 35% from carbohydrates. Both diets were obtained from Research Diets, Inc. (New Brunswick, NJ, USA).

### 2.25 Intestinal Organoid Culture

Intestinal crypts were isolated as previously described with minor modifications (Sato et al. 2009). Briefly, mouse small intestine was opened longitudinally, rinsed 10 times with DPBS, and cut into 0.5–1.0 cm fragments. The tissues were then incubated with 2.0 mM EDTA and 1.0 mg/mL collagenase II at 37°C for 20 min to dissociate the crypts. The supernatant containing crypts was filtered through a 70 µm strainer and centrifuged at 500 *g* for 5 min. Pellets were resuspended in culture medium, embedded in Matrigel, and seeded into pre‐warmed 96‐well plates, followed by polymerization for 10 min at 37°C and overlaying with 300 µL of culture medium. The medium consisted of advanced DMEM/F12 supplemented with glutamine, HEPES, penicillin/streptomycin, N2, B27, EGF, Wnt3a, Noggin, R‐spondin, nicotinamide, N‐acetylcysteine, SB202190, A83‐01, CHIR99021, and Y‐27632. Organoids were maintained at 37°C with 5% CO_2_, and the medium was refreshed every two days.

### 2.26 Construction of milRNA‐3020179 Knockout Strain of C. butyricum

The endogenous CRISPR‐Cas system targeting milRNA‐3020179 in *C. butyricum* was constructed as previously described (Zhang et al. 2020). Protospacer constructs carrying 5′ putative PAM sequences were generated by annealing primer pairs designed to produce EcoRI and BamHI cohesive ends for plasmid‐based interference assays. These constructs were subsequently cloned into the vector pMTL83151 between the EcoRI and BamHI sites to generate interference plasmids. Endogenous CRISPR array vectors were assembled following published protocols with minor modifications (Zhang et al. 2018, Zhou et al. 2021). Briefly, the synthetic CRISPR array was expressed under the control of a lactose‐inducible promoter derived from pJZ23‐Cas9 and terminated by a native CRISPR sequence from *C. tyrobutyricum*. The array contained a site‐specific 34‐nt spacer flanked by two identical 30‐nt direct repeats (5′‐GATTAACAGTAACATTAGATGTATTTAAAT‐3′). For gene editing, ∼700 bp upstream and downstream homology arms were amplified from the *C. butyricum* genome. The vectors, including pMTL83151 and pJZ23‐Cas9, were kindly provided by Jie Zhang from the Institute of Animal Science, Chinese Academy of Agricultural Sciences, and Wei Hong from Guizhou Medical University. To preliminarily assess potential polar effects and local transcriptional perturbations resulting from the knockout of milRNA‐3020179, the expression levels of three upstream and three downstream genes adjacent to milRNA‐3020179 in the KO *C. butyricum* strain were quantified by RT‐qPCR using primers listed in Table S5.

### 2.27 EV Treatment on Intestinal Organoids

To investigate the effects of EVs derived from wild‐type (WT) and knockout strains of *C. butyricum* on lipid metabolism, cells were treated with 50 µg protein of EVs for 24 h.

### 2.28 Immunofluorescence Analysis

The intestinal organoids were washed three times with ice‐cold HBSS and subsequently resuspended in cold HBSS. The organoids were centrifuged at 300 *g* for 10 min at 4°C and then fixed overnight in 4% paraformaldehyde. After fixation, the samples were permeabilized with 0.5% Triton X‐100 for 20 min, washed three times with HBSS, and blocked with 5% bovine serum albumin in HBSS for 1 h to minimize nonspecific staining. For immunofluorescence staining, the organoids were incubated overnight at 4°C with primary antibodies against PPARα (1:50, Abcam) and RXRα (1:50, Bioss). After washing, the samples were incubated with goat anti‐rabbit DyLight 594‐conjugated secondary antibodies (1:200, Abcam) for 1 h, followed by DAPI staining for 5 min at 25°C. Fluorescent images were acquired using a Zeiss LSM‐710 laser‐scanning confocal microscope.

### 2.29 Statistics Analysis

Statistical analyses were performed using SPSS Statistics (version 22.0) and GraphPad Prism (version 9.0). One‐way analysis of variance (ANOVA) followed by the Student‐Newman‐Keuls post hoc test or a two‐tailed Student's *t*‐test was applied where appropriate. All tests were two‐sided and data are expressed as mean ± SEM. Differences were considered statistically nonsignificant at P > 0.05. Statistical significance was set at P < 0.05, and high significance levels were indicated at P < 0.01, and P < 0.001. The statistical tests used for each experiment and the details regarding experimental reproducibility were specified in the figure legends. No data were excluded from the analysis. ImageJ software (version 1.53; National Institutes of Health, USA) was used for densitometric analysis of Western blot results.

## 3 Results

### 3.1 Crude Fat Digestibility Is Correlated With Gut Microbiota Composition

Apparent crude fat digestibility reflects the host's capacity for dietary fat metabolism. We first evaluated apparent crude fat digestibility in weaned piglets. Based on the results, the ten piglets with the highest digestibility were assigned to the HIGH group, while the ten piglets with the lowest digestibility were assigned to the LOW group (Figure 1A). Compared with the LOW group, HIGH piglets exhibited reduced fecal TG and NEFA concentrations (Figure 1B‐C) but elevated serum TG and NEFA levels (Figure 1D‐E). They also showed higher bile acid concentrations and lipase activity in ileal contents, accompanied by increased serum levels of SCT and CCK (Figure S2A‐D). At the molecular level, ileal tissues from HIGH piglets displayed markedly upregulated expression of cluster of differentiation 36 (*CD36*), fatty acid transport protein 4 (*FATP4*), and fatty acid binding protein 2 (*FABP2*), which are key mediators of lipid transport (Figure 1F). Moreover, genes associated with lipid synthesis and oxidation, including fatty acid synthase (*FASN*), stearoyl‐CoA desaturase (*SCD*), and carnitine palmitoyltransferase 1A (*CPT1A*), were significantly upregulated in the ileum (Figure 1G), whereas no such changes were detected in the jejunum (Figure S1E‐F). Consistent with these transcriptional alterations, fatty acid oxidation (FAO) activity was significantly higher in the ileum of HIGH piglets (Figure 1H). Collectively, these findings demonstrate that piglets with higher crude fat apparent digestibility are characterized by enhanced fat digestion, transport, and oxidation.

**FIGURE 1:**
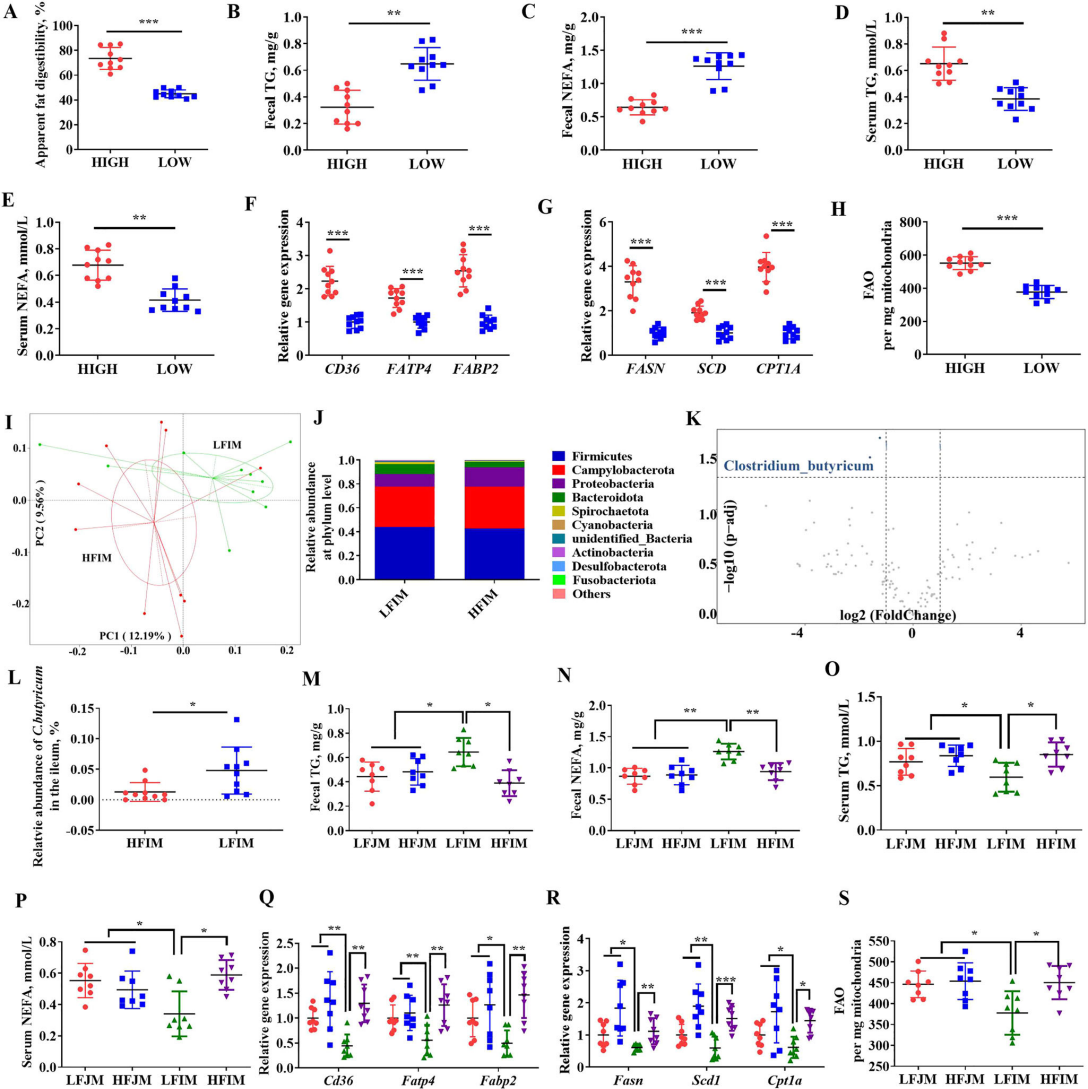
Crude fat digestibility is correlated with gut microbiota composition (A) Apparent fat digestibility; Fecal TG (B) and NEFA (C) contents; Serum TG (D) and NEFA (E) contents; (F) Relative expression of genes involved in lipid transport; (G) Relative expression of genes involved in fat oxidation and synthesis; (H) FAO activity; (I) PCoA based on unweighted UniFrac distance; (J) Relative abundance of ileal mucosa microbiota at the phylum level; (K) The volcano plots of the differential microbes; (L) Relative abundance of *C. butyricum* in the ileum mucosa via RT‐qPCR; (Data were analyzed by two‐tailed Student's *t*‐test, n = 10). Fecal TG (M) and NEFA (N) contents, and serum TG (O) and NEFA (P) contents in mice treated with mucosa microbiota transplantation; (Q) Relative expression of genes involved in lipid transport; (R) Relative expression of genes involved in fat oxidation and synthesis; (S) FAO activity. (Data were analyzed by one‐way ANOVA, n = 8). Values are presented as mean ± SEM. ^*^ *P* < 0.05. ^**^ *P* < 0.01. ^***^ *P* < 0.001.

We next conducted 16S rRNA sequencing to examine gut microbiota composition in piglets with different levels of crude fat apparent digestibility. Overall, no significant differences were observed in α‐ or β‐diversity across microbial communities from various intestinal contents or mucosal sites between the HIGH and LOW groups (Figure S3), except for a distinct difference in β‐diversity of the ileal mucosal microbiota (Figure 1I). At the phylum level, the relative abundance of Firmicutes was comparable between groups (Figure 1J). However, at the species level, *C. butyricum* was significantly less abundant in the ileal mucosa of HIGH piglets compared with LOW piglets (Figure 1K), a finding further validated by fecal microbial quantification using RT‐qPCR (Figure 1L). No other significant microbial differences were detected at corresponding intestinal sites (Figure S4).

To determine whether mucosal microbiota influence fat digestibility, jejunal and ileal mucosal microbiota from donor piglets were transplanted into recipient mice. Mice colonized with ileal mucosal microbiota from LOW piglets (LFIM) exhibited elevated fecal TG and NEFA concentrations and reduced serum TG and NEFA levels compared with those colonized with ileal mucosal microbiota from HIGH piglets (HFIM) or jejunal mucosal microbiota from either group (LFJM and HFJM) (Figure 1M‐P). Hepatic TG and NEFA levels did not differ among groups (Figure S5). Notably, LFIM mice displayed decreased ileal expression of lipid metabolism‐related genes, including *Fasn*, *Cpt1a*, *Scd1*, *Cd36*, *Fatp4*, and *Fabp2* (Figure 1Q‐R), along with reduced FAO activity (Figure 1S), relative to other groups. Collectively, these findings demonstrate that ileal mucosal microbiota play a pivotal role in regulating intestinal lipid metabolism.

### 3.2 C. butyricum Reduces Intestinal Lipid Absorption and Oxidation

We next investigated whether the abundance of *C. butyricum* in the ileal mucosa contributes to intestinal lipid metabolism. A strain of *C. butyricum* was isolated from pigs and orally administered to mice. Colonization of *C. butyricum* in the ileum was confirmed by its isolation and culture from the ileal mucosa. Subsequently, we found that supplementation with *C. butyricum* significantly decreased serum TG, NEFA, and TC levels (Figure 2A‐C), while increasing their fecal concentrations (Figure 2D‐F). In addition, *C. butyricum* reduced FAO activity in the ileum (Figure 2G), but not in the jejunum (Figure 2H). Both the TG tolerance test and inhibition of lipoprotein lipase (LPL) further confirmed that *C. butyricum* lowered post‐gavage serum TG levels (Figure 2I‐J). These results indicate that *C. butyricum* suppresses ileal lipid absorption and oxidation. However, no significant changes were detected in fecal bile acid content, ileal lipase activity, or serum SCT and CCK concentrations (Figure S6), suggesting that *C. butyricum* does not affect lipid digestion directly.

**FIGURE 2:**
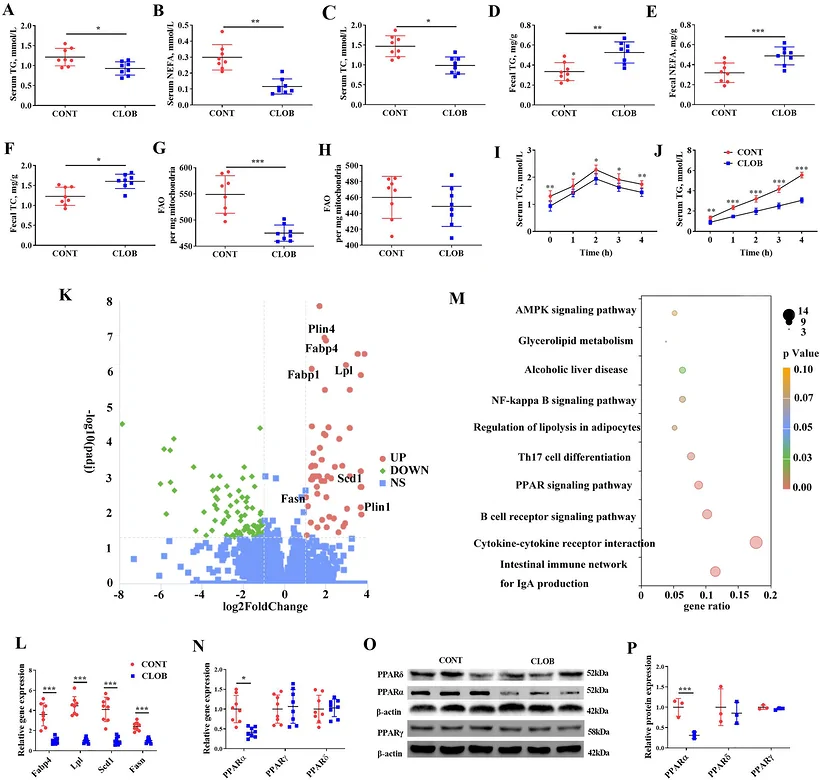
*C. butyricum* reduces intestinal lipid absorption and oxidation Serum TG (A), NEFA (B) and TC (C) contents; Fecal TG (D), NEFA (E) and TC (F) contents; FAO activity in the ileum (G) and jejunum (H); (I) TG tolerance test; (J) LPL inhibition test; (K) The volcano plots of the differential genes (CONT vs CLOB); (L) Relative abundance of genes involved in lipid metabolism via RT‐qPCR; (M) KEGG enrichment analysis; (N) Relative expression of *PPAR* genes; (O, P) Relative expression of PPAR proteins (O, Western blotting results; P, qualification); (Data were analyzed by two‐tailed Student's *t*‐test or one‐way ANOVA, n = 8 or n = 3). Values are presented as mean ± SEM. ^*^ *P* < 0.05. ^**^ *P* < 0.01. ^***^ *P* < 0.001.

To further investigate the impact of *C. butyricum* on host intestinal function, we conducted transcriptome profiling of ileal tissues. A total of 145 genes were differentially expressed between control mice and *C. butyricum*‐treated mice, including 59 upregulated and 86 downregulated genes. Notably, several lipid metabolism‐related genes, such as *Fabp1*, *Fabp4*, *Lpl*, *Plin1*, *Plin4*, *Scd1*, and *Fasn*, were significantly downregulated following *C. butyricum* administration (Figure 2K). Among these, reductions in *Fabp4*, *Lpl*, *Scd1*, and *Fasn* were further validated by RT‐qPCR (Figure 2L). KEGG enrichment analysis indicated that those differentially expressed genes were enriched in lipid metabolism‐associated pathways, including glycerolipid metabolism, regulation of lipolysis, and the PPAR signaling pathway (Figure 2M). Given the central role of PPAR signaling in intestinal lipid metabolism, we next examined the expression of *PPARα*, *PPARγ*, and *PPARδ*. RT‐qPCR analysis revealed that *C. butyricum* specifically downregulated *PPARα* expression, with no significant effects on *PPARγ* or *PPARδ* (Figure 2N). This result was further supported at the protein level, where a reduction in PPARα, but not PPARγ or PPARδ, was observed (Figure 2O‐P). Together, these findings indicate that *C. butyricum* suppresses intestinal lipid absorption and oxidation primarily through inhibition of PPARα signaling.

### 3.3 C. butyricum Regulates Lipid Absorption and Oxidation via EV‐encapsulated Small RNAs

It is generally assumed that the beneficial effects of *C. butyricum* are primarily mediated by butyrate production. To test this hypothesis, we administered sodium butyrate to mice. However, no significant differences were detected in serum or fecal concentrations of TG, NEFA, and TC (Figure S7A‐F), nor in the ileal expression of genes associated with lipid transport, fatty acid synthesis, or oxidation (Figure S7G‐H).

Given that gut microbes can influence host physiology not only through metabolites but also via EVs, we next isolated and purified EVs from *C. butyricum*. TEM revealed their characteristic saucer‐shaped morphology (Figure 3A), while NTA indicated an average vesicle diameter of 77.8 nm (Figure 3B). Moreover, EV‐associated proteins including GroEL and GAPDH were detected in the EVs (Figure 3C). The DiR‐labeled EVs were then orally administered to mice, and in vivo imaging confirmed their localization in the intestine (Figure S8). Functionally, treatment with EVs significantly decreased serum TG, NEFA, and TC levels (Figure 3D), while elevating their concentrations in feces (Figure 3E). In the ileum, EV exposure led to downregulation of Lpl, Cd36, Fatp4, and Fabp2 expression at both the mRNA and protein levels (Figure 3F‐H). Furthermore, EVs suppressed the expression of PPARα and its dimerization partners Rxra at both transcriptional and translational levels (Figure 3I‐K). Consistent with reduced PPARα activity, EV treatment diminished ileal FAO activity (Figure 3L), as well as lipid absorption, as evidenced by the TG tolerance test and LPL inhibitor assay (Figure 3M‐N). Collectively, these results demonstrate that *C. butyricum*‐derived EVs inhibit ileal lipid absorption and oxidation.

**FIGURE 3:**
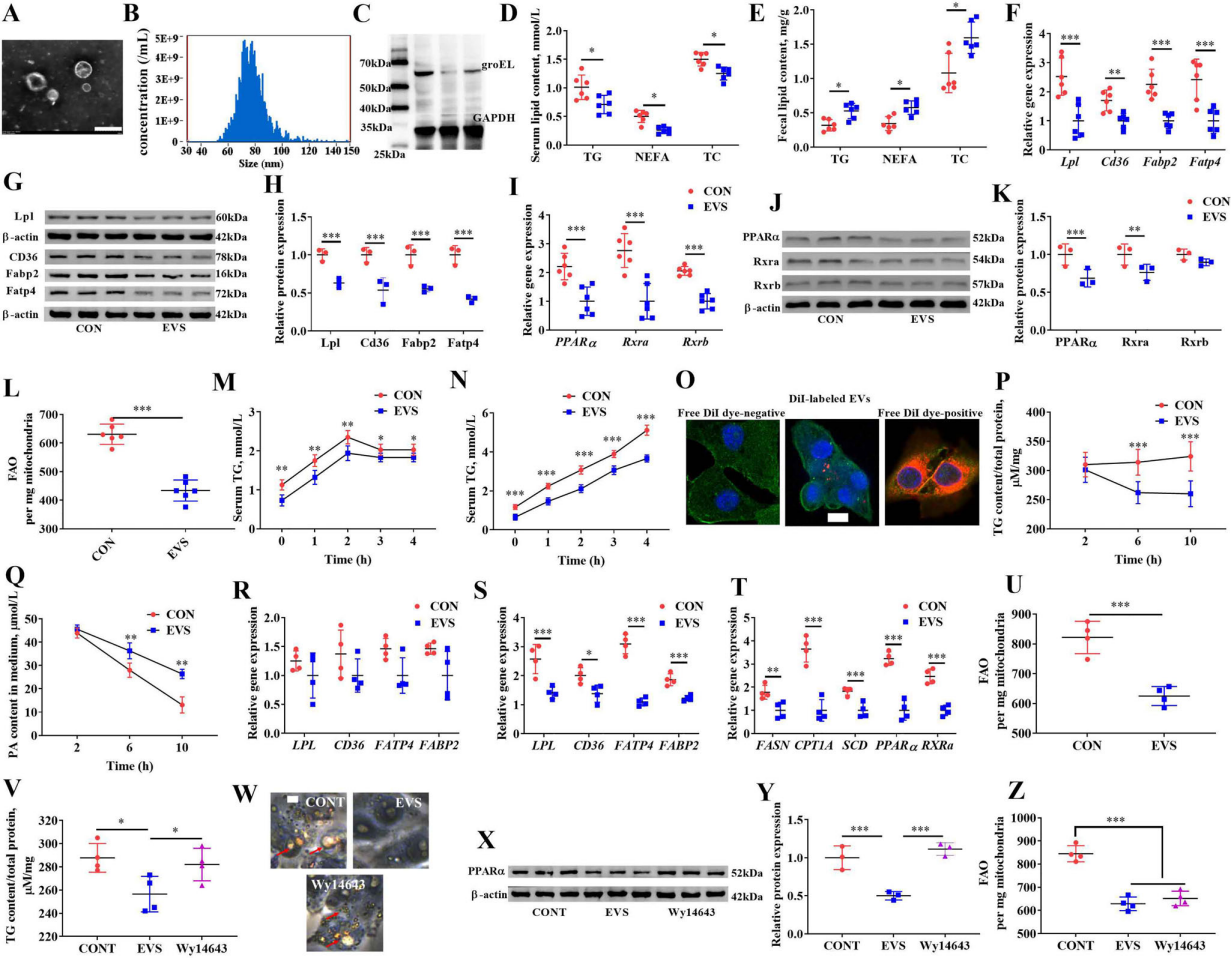
*C. butyricum* regulates lipid absorption and oxidation via EV‐encapsulated small RNAs (A) *C. butyricum*‐derived EVs observed under transmission electron microscopy (scale bar = 200 nm); (B) EV size detected by nanoparticle tracking analysis; (C) Expression of GroEL and GAPDH proteins in EVs; (D) Serum lipid content; (E) Fecal lipid content; (F) Expression of genes involved in lipid metabolism; (G, H) Expression of proteins involved in lipid metabolism (G, Western blotting results; H, qualification); (I) Relative expression of *PPAR* and *Rxr* genes; (J, K) Relative expression of PPAR proteins (J, Western blotting results; K, qualification); (L) FAO activity in the ileum; (M) TG tolerance test; (N) LPL inhibition test; (Data were analyzed by two‐tailed Student's *t*‐test or one‐way ANOVA, n = 6 or n = 3). (O) EVs entered into intestinal epithelial cells (Left, IPEC‐J2 cells treated with free DiI dye processed using the same purification procedure in the absence of EVs; Middle, IPEC‐J2 cells treated with purified EVs labeled with DiI dye; Right, IPEC‐J2 cells treated with free DiI dye. Blue, DAPI; Red, DiI‐labeled EVs; Green, β‐actin; scale bar = 10 µm); (P) TG content in IPEC‐J2 cells; (Q) PA content in the culture medium; Relative expression of genes involved in lipid transport after 2 h (R) and 6 h (S) treated with *C. butyricum*‐derived EVs; (T) Relative expression of genes involved in lipid metabolism after 2 h treated with EVs; (U) FAO activity in IPEC‐J2 cells; (V) TG content in IPEC‐J2 cells; (W) Lipid droplets in IPEC‐J2 cells detected by Oil Red O staining (red arrow, lipid droplet; scale bar = 15 µm); (X, Y) Relative expression of PPARα proteins (X, Western blotting results; Y, qualification); (Z) FAO activity. (Data were analyzed by two‐tailed Student's *t*‐test or one‐way ANOVA, n = 4 or n = 3). Values are presented as mean ± SEM. ^*^ *P* < 0.05. ^**^ *P* < 0.01. ^***^ *P* < 0.001.

To further explore the underlying mechanisms, we initially assessed cell viability and found that treatment with EVs did not induce cytotoxicity in IPEC‐J2 cells (Figure S9). Then, we treated IPEC‐J2 cells with 50 µM palmitic acid (PA) in the presence or absence of EVs. Confocal imaging showed that an average of 63% of the cells exhibited DiI‐positive signals, suggesting the uptake of EVs by intestinal epithelial cells (Figure 3O). Importantly, this observation was not attributable to EV‐induced cytotoxicity or membrane damage, as demonstrated by the cell viability assay. EV treatment reduced intracellular lipid accumulation (Figure 3P) and delayed PA depletion from the culture medium (Figure 3Q) at both 6 and 10 h. Gene expression analysis showed that *LPL*, *CD36*, *FATP4*, and *FABP2* were unaffected at 2 h but significantly downregulated at 6 h (Figure 3R‐S). In contrast, expression of *FASN*, *CPT1A*, *SCD*, *PPARα*, and *RXRa*, as well as FAO activity, was already decreased at 2 h (Figure 3T‐U). Importantly, co‐treatment with the PPARα agonist Wy14643 reversed the effects of EVs on lipid accumulation (Figure 3V‐W, Figure S10) and PPARα expression (Figure 3X‐Y), although it failed to rescue FAO activity at 6 h (Figure 3Z). These results indicate that EVs suppress FAO directly while concurrently reducing lipid uptake through downregulation of PPARα signaling.

To identify the active components of EVs, we pretreated vesicles with RNase A, DNase, or proteinase K before co‐culture with IPEC‐J2 cells. First, EVs were subjected to treatment with RNase A, Triton X‐100, a combination of RNase A and Triton X‐100, or RNase A buffer plus Triton X‐100. The results demonstrated that milRNA‐3020179 were degraded only upon combined treatment with RNase A and Triton X‐100 (Figure S11). Then, we found that EVs treated with DNase or proteinase K retained their inhibitory effects on lipid accumulation, FAO activity, and the expression of lipid metabolism‐related genes (*LPL*, *CD36*, *FATP4*, *FABP2*, *FASN*, *CPT1A*, *SCD*, *PPARα*, *RXRa*). In contrast, RNase A pretreatment completely abolished these effects (Figure S12). Together, these findings demonstrate that small RNAs encapsulated within *C. butyricum*‐derived EVs are the primary mediators driving the regulation of intestinal lipid metabolism. Furthermore, RNAs <200 nt were isolated from EVs and fractionated into 20–40 nt and >40 nt groups. Only the 20–40 nt fraction effectively suppressed FAO activity and the expression of lipid metabolism‐related genes, whereas RNAs of 40–200 nt had no significant effect (Figure S13).

### 3.4 MilRNAs Derived From C. butyricum Regulate Intestinal Lipid Metabolism

To further identify specific small RNAs involved in the regulation of lipid metabolism, we performed small RNA sequencing. Total and clean read counts were obtained, and the clean reads were subsequently mapped to the *C. butyricum* reference genome. Reads of 18–35 nt in length were analyzed, and both total and unique reads encapsulated within EVs were quantified, with their length distribution presented as percentages (Figure 4A, Figure S14). Among these small RNAs, three novel miRNA‐like RNAs (milRNA‐409778, milRNA‐3020179, and milRNA‐3047216) were consistently detected across all four biological replicates. Their expression was further validated by qPCR and confirmed by agarose gel electrophoresis (Figure 4B). To predict potential host targets, we applied the Miranda algorithm. The results indicated that milRNA‐409778 may target *Rxra* and *Rxrb* in mice and *FASN* and *CPT1A* in pigs; milRNA‐3020179 may target *PPARα* and *Rxra* in mice and pigs; and milRNA‐3047216 may target *Lpl* in mice and *PLIN1* and *SCD* in pigs (Figure 4C).

**FIGURE 4:**
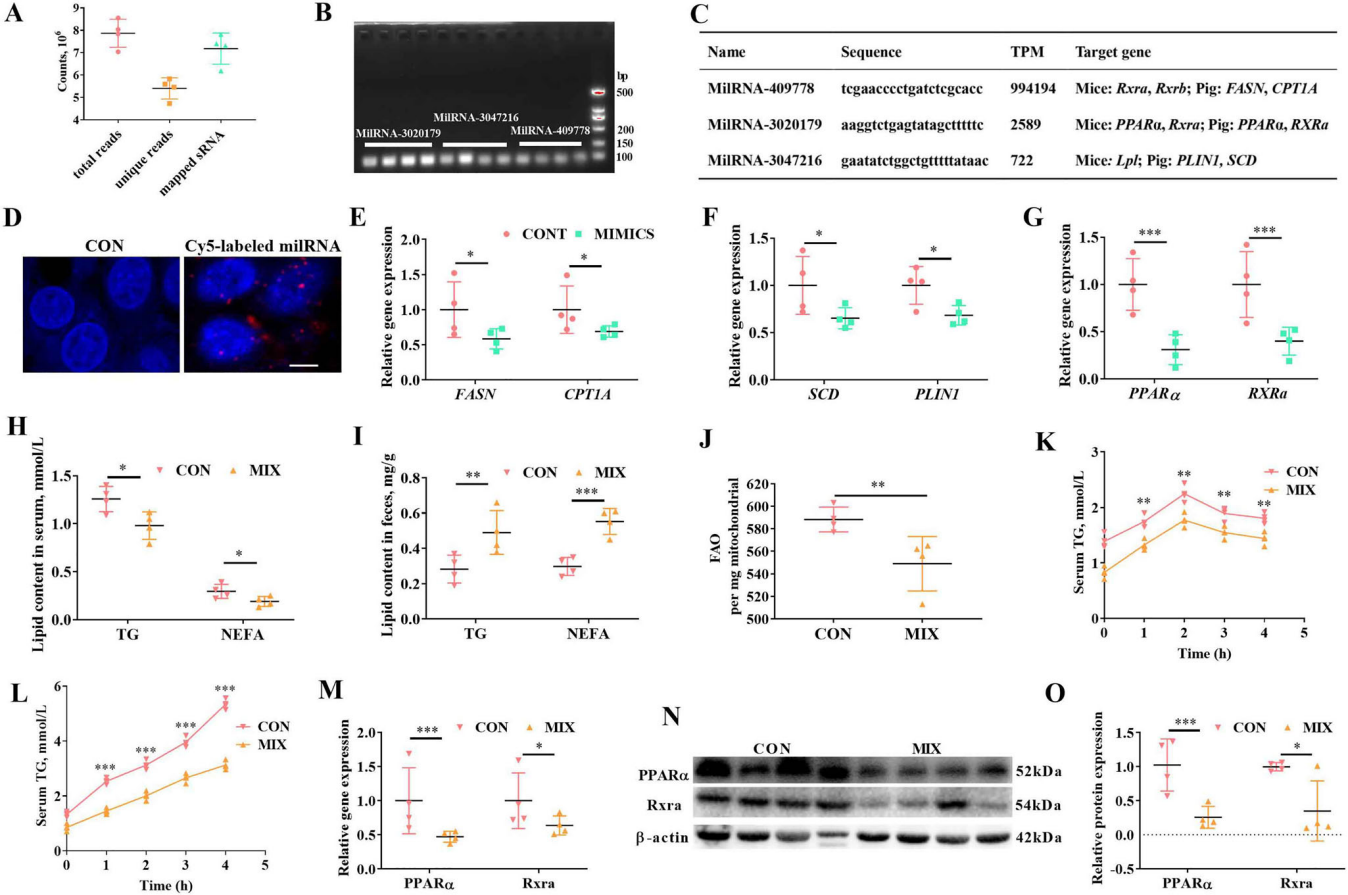
MilRNAs derived from *C. butyricum* regulate intestinal lipid metabolism (A) Total, unique and mapped reads encapsulated within *C. butyricum*‐derived EVs; (B) Validation of milRNAs expressed in EVs by RT‐qPCR and agarose gel electrophoresis; (C) Target genes of milRNAs; (D) MilRNAs being taken up by IPEC‐J2 cells (Red, Cy5‐labeled milRNA; Blue, DAPI; scale bar = 5 µm). CON, cells treated with transfection reagent alone; Cy5‐labeled milRNA, cells treated with transfection reagent and Cy5‐labeled milRNA‐3020179. (E) Expression of target genes of milRNA‐409778; (F) Expression of target genes of milRNA‐3047216; (G) Expression of target genes of milRNA‐3020179; Lipid content in serum (H) and feces (I); (J) FAO activity; (K) TG tolerance test; (L) LPL inhibition test; (M) Relative expression of *PPARα* and *Rxra*; (N, O) Relative expression of PPARα and Rxra proteins (N, Western blotting results; O, qualification). (Data were analyzed by two‐tailed Student's *t*‐test or one‐way ANOVA, n = 4). Values are presented as mean ± SEM. ^*^ *P* < 0.05. ^**^ *P* < 0.01. ^***^ *P* < 0.001.

We next synthesized Cy5‐labeled milRNAs and confirmed their uptake by IPEC‐J2 cells (Figure 4D, Figure S15). The cells were subsequently treated with mimics of milRNA‐409778, milRNA‐3020179, or milRNA‐3047216. Co‐culture with milRNA‐409778 mimics resulted in decreased expression of *FASN* and *CPT1A* (Figure 4E), while milRNA‐3047216 mimics reduced *SCD* and *PLIN1* expression (Figure 4F), and milRNA‐3020179 mimics downregulated *PPARα* and *RXRa* expression (Figure 4G). Next, mice were administered agomirs separately or mixtures corresponding to milRNA‐409778, milRNA‐3020179, and milRNA‐3047216. Analysis of ileal samples confirmed that agomir treatment significantly increased the expression levels of milRNA‐409778, milRNA‐3020179, and milRNA‐3047216 (Figure S16). Notably, administration of the milRNA mixture reduced serum lipid levels (Figure 4H) but increased fecal lipid excretion (Figure 4I). Moreover, the milRNA treatment suppressed FAO activity and lipid uptake (Figures 4J‐L) and inhibited the PPARα signaling pathway, as evidenced by reduced PPARα and Rxra expression in the ileum (Figures 4M‐O). Collectively, these findings demonstrate that milRNA‐409778, milRNA‐3020179, and milRNA‐3047216, which were encapsulated within *C. butyricum*‐derived EVs, modulate host intestinal lipid metabolism.

### 3.5 C. butyricum and Its Derived EVs Attenuate HFD‐induced Lipid Deposition in Mice

To investigate whether *C. butyricum* and its derived EVs could influence the development of HFD‐induced lipid metabolic alterations, mice were simultaneously subjected to HFD feeding and treatment with either *C. butyricum* or its EVs. Both treatments attenuated HFD‐associated body weight gain and reduced inguinal and epididymal fat mass (Figures 5A–5B). Liver weight and hepatic TG accumulation were also lower in mice receiving *C. butyricum* or EVs (Figure 5C). In addition, serum TG and NEFA concentrations were decreased (Figure 5D), whereas their levels in feces were increased (Figure 5E). Ileal TG content was likewise reduced following administration of *C. butyricum* or its EVs (Figures 5F–5G). Consistent with these observations, the expression of genes involved in lipid uptake and transport, including *Fatp4*, *Lpl*, *Fabp2*, and *Cd36*, was significantly downregulated (Figure 5H). Expression of *Cpt1a* was also decreased (Figure 5I), accompanied by lower gene and protein expression of PPARα and Rxra (Figures 5J–L). Furthermore, TG tolerance tests and treatment with an LPL inhibitor supported an association between *C. butyricum* or EV supplementation and reduced intestinal lipid uptake (Figures 5M–5N). Collectively, these findings indicate that concurrent administration of *C. butyricum* or its derived EVs during HFD feeding attenuates the development of lipid accumulation and is associated with reduced intestinal lipid absorption.

**FIGURE 5:**
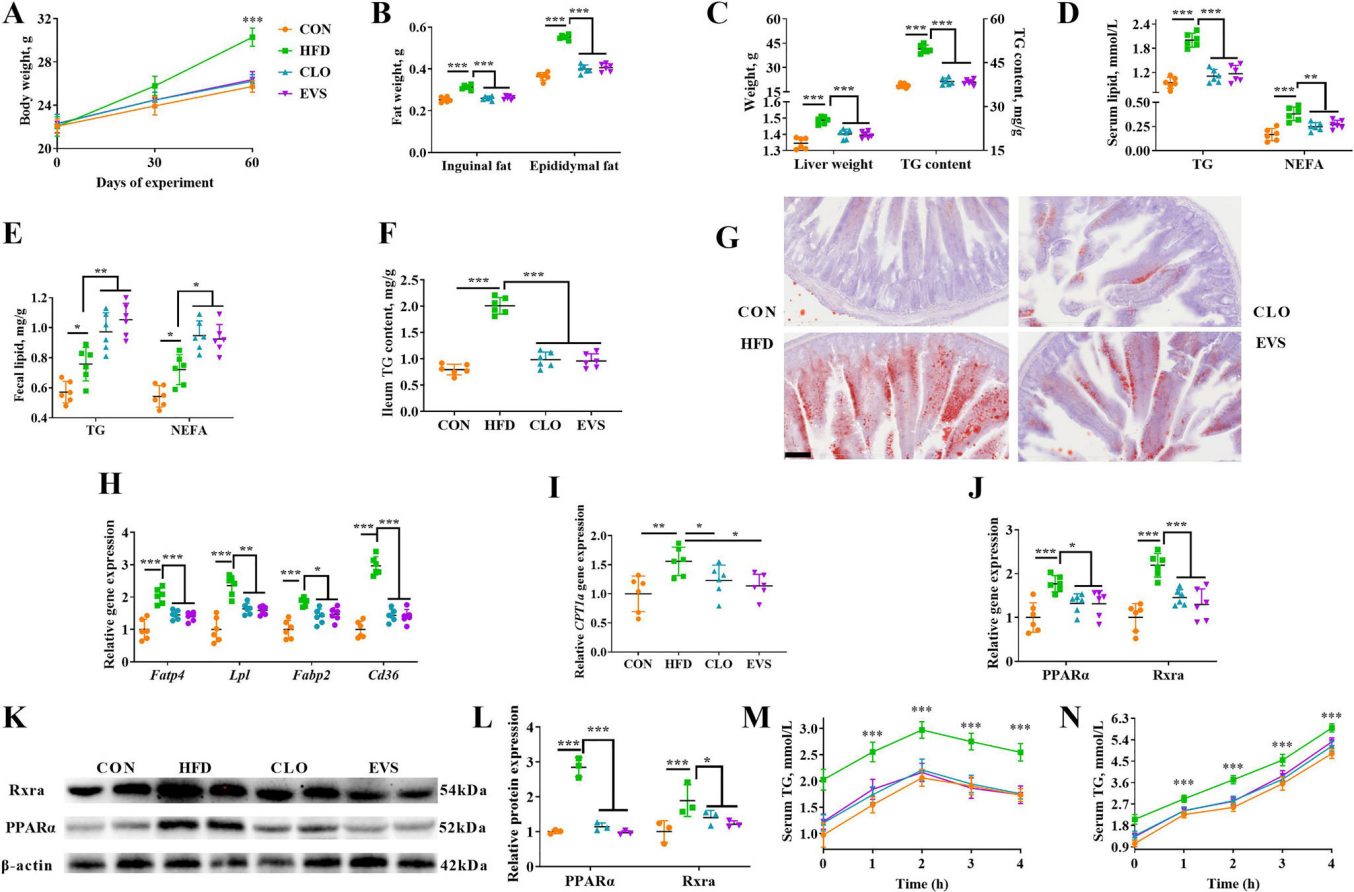
*C. butyricum* and its derived EVs protects against lipid deposition in HFD‐fed mice (A) Body weight; (B) Fat weight; (C) Liver weight and TG content; (D) Serum lipid content; (E) Fecal lipid content; (F) Ileum TG content; (G) Lipid droplets detected by Oil Red O staining (Red, lipid droplet; scale bar = 100 µm); (H) Relative expression of genes involved in lipid transport in the ileum; (I) Relative *Cpt1a* gene expression; (J) Relative expression of *PPARα* and *Rxra*; (K, L) Relative expression of PPARα and Rxra proteins (K, Western blotting results; L, qualification); (M) TG tolerance test; (N) LPL inhibition test. (Data were analyzed by one‐way ANOVA, n = 6 or n = 3). Values are presented as mean ± SEM. ^*^ *P* < 0.05. ^**^ *P* < 0.01. ^***^ *P* < 0.001.

### 3.6 C. butyricum Regulates Lipid Metabolism via Its Derived milRNA in Intestinal Organoids

To further investigate whether *C. butyricum* regulates PPARα signaling and intestinal lipid metabolism through its derived milRNAs, we generated a milRNA‐3020179 knockout strain of *C. butyricum* (CLO‐KO) using the CRISPR/Cas9 system. Sequencing analysis confirmed complete deletion of the target sequence (Figure S17), and milRNA‐3020179 expression was undetectable in EVs derived from the CLO‐KO strain (Figure S18). Furthermore, no significant differences were observed in the growth curve or in the expression levels of three upstream and three downstream genes adjacent to milRNA‐3020179 in the KO *C. butyricum* strain, suggesting that the knockout of milRNA‐3020179 does not appear to induce detectable polar effects or local transcriptional perturbations under the conditions examined (Figure S19). Then, mouse‐derived intestinal organoids were pretreated with PA and then cocultured with CLO‐KO, while *Lactobacillus rhamnosus* GG (LGG) was included as a control to assess whether other microbes exerted similar effects. *C. butyricum* treatment significantly decreased TG content in organoids (Figures 6A) while increasing PA levels in the culture medium (Figures 6B), whereas CLO‐KO and LGG showed no such effects. These findings indicate that deletion of milRNA‐3020179 impairs the ability of *C. butyricum* to modulate intestinal lipid absorption. Consistently, analysis of proteins involved in lipid oxidation and transport revealed that CLO‐KO did not significantly alter CPT1a, CD36, or Fatp4 expression, in contrast to *C. butyricum* (Figures 6C‐D). However, both CLO‐KO and *C. butyricum* reduced Lpl protein levels in organoids (Figures 6C‐D). At the transcriptional level, *C. butyricum* markedly downregulated *PPARα*, *Rxra*, and *Lpl* expression, whereas CLO‐KO decreased *Rxra* and *Lpl* but had no effect on *PPARα* (Figure 6E). Immunofluorescence staining further demonstrated that *C. butyricum* suppressed PPARα and Rxra expression, while LGG had no effect. In contrast, CLO‐KO reduced Rxra levels without affecting PPARα expression (Figure 6F). Collectively, these results suggest that *C. butyricum* regulates PPARα signaling through its derived milRNA‐3020179, whereas other milRNAs, such as milRNA‐409778 and milRNA‐3047216, may additionally target Rxra and Lpl, respectively.

**FIGURE 6:**
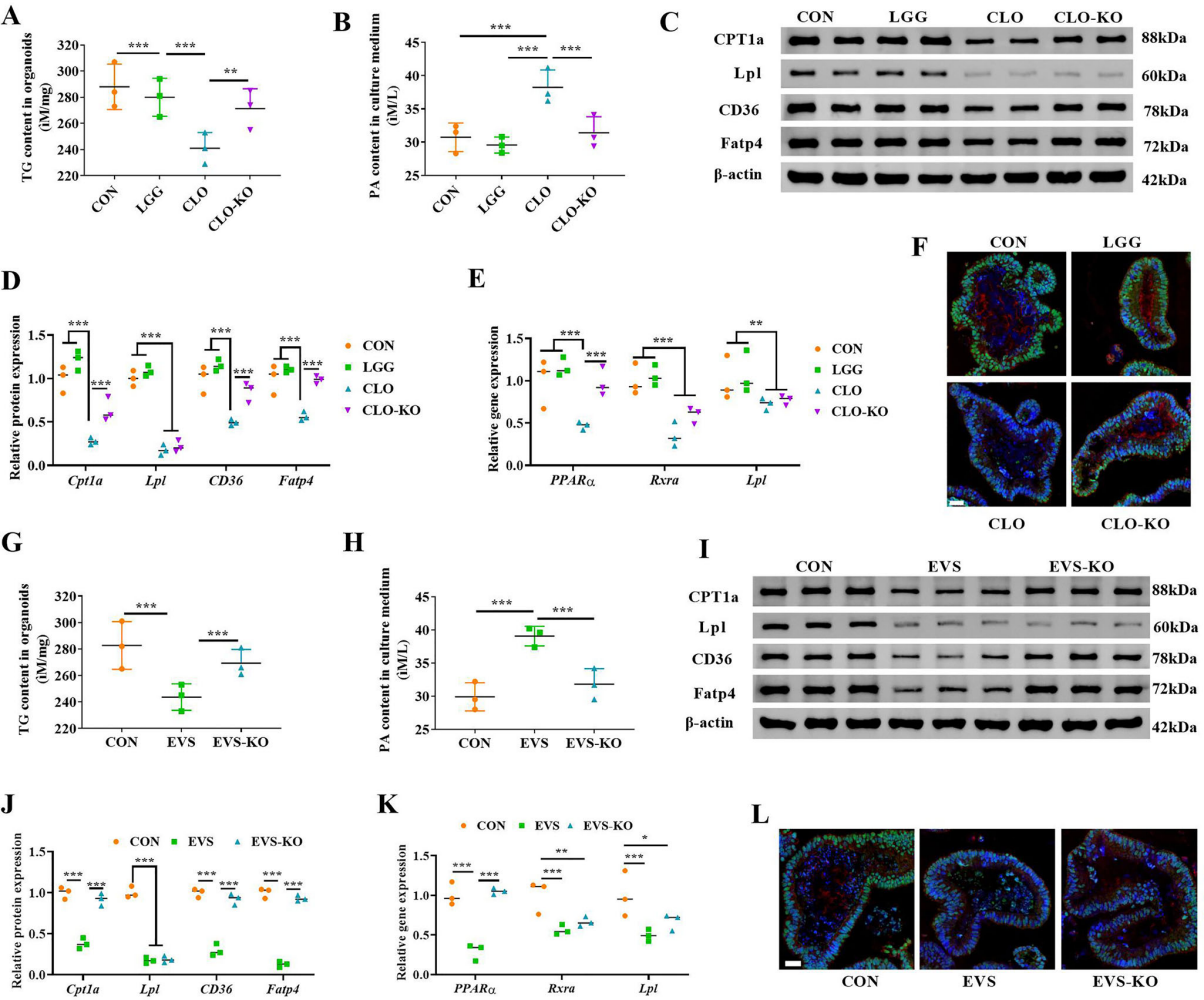
*C. butyricum* regulates lipid metabolism via its derived milRNA in intestinal organoids (A) TG content in organoids treated with milRNA‐3020179 knockout strain of *C. butyricum*; (B) PA content in the culture medium; (C, D) Relative expression of proteins involved in lipid metabolism (C, Western blotting results; D, qualification); (E) Relative expression of genes; (F) Protein expression detected by immunofluorescence staining (Green, Rxra; Red, PPARα; Blue, DAPI; scale bar = 25 µm); (G) TG content in organoids treated with EVs from milRNA‐3020179 knockout strain of *C. butyricum*; (H) PA content in the culture medium; (I, J) Relative expression of proteins involved in lipid metabolism (I, Western blotting results; J, qualification); (K) Relative expression of genes; (L) Protein expression detected by immunofluorescence staining (Green, Rxra; Red, PPARα; Blue, DAPI; scale bar = 25 µm). (Data were analyzed by one‐way ANOVA, n = 3). Values are presented as mean ± SEM. ^*^ *P* < 0.05. ^**^ *P* < 0.01. ^***^ *P* < 0.001.

To further determine whether *C. butyricum* regulates PPARα signaling via milRNAs encapsulated in its EVs, we isolated EVs from WT and knockout strains. EVs from WT *C. butyricum* significantly decreased TG content in colonic organoids (Figures 6G) while increasing PA levels in the culture medium (Figures 6H), whereas EVs from the CLO‐KO strain had no such effects. Similarly, analysis of lipid oxidation and transport proteins showed that EVs from CLO‐KO did not significantly alter CPT1a, CD36, or Fatp4 expression, unlike EVs from WT *C. butyricum* (Figures 6I‐J). Both WT and CLO‐KO EVs reduced Lpl protein levels in the organoids (Figures 6I‐J). At the transcriptional level, EVs from WT *C. butyricum* markedly downregulated *PPARα*, *Rxra*, and *Lpl* expression, whereas EVs from CLO‐KO decreased *Rxra* and *Lpl* but had no effect on PPARα (Figure 6K). Consistently, immunofluorescence analysis revealed that EVs from WT *C. butyricum* significantly reduced PPARα and Rxra expression, while EVs from CLO‐KO only decreased Rxra without affecting PPARα (Figure 6L). Together, these findings confirm that milRNA‐3020179, encapsulated within *C. butyricum*‐derived EVs, inhibits intestinal lipid absorption by targeting the PPARα signaling pathway. In contrast, other milRNAs, such as milRNA‐409778 and milRNA‐3047216, might downregulate genes including *Rxra* and *Lpl* but do not influence lipid absorption.

## 4 Discussion

Accumulating evidence underscores the pivotal role of intestinal microbes in regulating lipid metabolism. Notably, diseases associated with dyslipidemia, such as non‐alcoholic fatty liver disease and obesity, are often accompanied by alterations in gut microbiota composition (Jayachandran and Qu 2023, Van Hul and Cani 2023). Intestinal microbes modulate lipid metabolism through diverse direct and indirect mechanisms, primarily via their metabolites, including short‐chain fatty acids and bile acids, as well as microbial components such as lipopolysaccharides (Ghazalpour et al. 2016, Schoeler and Caesar 2019). Recently, EVs derived from gut microbes have gained increasing attention as key mediators of microbial effects. In our study, we first observed that microbes colonizing the proximal ileal mucosa was closely associated with intestinal lipid uptake. Notably, a strain of *C. butyricum* isolated from the pigs significantly reduced lipid absorption. Previous studies have demonstrated the therapeutic potential of *C. butyricum* in established HFD‐induced obesity (Liao et al. 2023, Seo et al. 2013, Zhou et al. 2017). In contrast, the present study was designed to evaluate its preventive effects on the development of HFD‐induced metabolic disturbances. Accordingly, HFD feeding and treatment administration were initiated simultaneously, and the findings indicate that *C. butyricum* attenuates the progression of metabolic alterations during HFD exposure. Mechanistically, *C. butyricum* attenuated lipid uptake and limited the development of HFD‐induced lipid accumulation in both intestinal organoids and mouse models via milRNAs encapsulated in its EVs, which directly targeted PPARα.

*C. butyricum* is a probiotic widely recognized for its ability to inhibit lipid accumulation, thereby preventing obesity and fatty liver (Liao et al. 2023, Seo et al. 2013, Zhou et al. 2017, Liao et al. 2023). However, the underlying mechanisms remain incompletely understood. Accumulating evidence suggests that *C. butyricum* influences host lipid metabolism through intestinal pathways. Although butyrate‐producing bacteria are typically enriched in the cecum and colon due to the strictly anaerobic environment and the abundance of fermentable substrates (Louis and Flint 2017), low‐level colonization and metabolic activity of these bacteria have also been detected in the ileum (Handa et al. 2023, Hoogeveen et al. 2020), suggesting that *C. butyricum* may exert functional effects in this region as well. Butyrate, the major metabolite of *C. butyricum*, is considered the key mediator of its regulatory effects on lipid metabolism (Li et al. 2022). Nevertheless, other studies have reported that *C. butyricum* reduces obesity through butyrate‐independent mechanisms. For instance, C. butyricum may suppress lipogenesis via bacterial cell wall components and by altering gut microbiota composition and metabolism, including tryptophan and purine metabolic pathways (Liao et al. 2023, Liao et al. 2023). However, the specific wall components involved and the precise mechanisms by which *C. butyricum* reshapes gut microbiota metabolism remain unclear. In this study, we found that higher *C. butyricum* abundance was correlated with lower fat digestibility, consistent with previous findings that Jinhua pigs with lower fatness harbor higher levels of *C. butyricum* (Ma et al. 2024). Moreover, administration of *C. butyricum* reduced intestinal lipid absorption. Importantly, EVs derived from *C. butyricum*, rather than its metabolite butyrate, played a central role. We further demonstrated that specific milRNAs encapsulated in these EVs precisely targeted the PPARα pathway. Collectively, our results provide insights into the mechanisms by which *C. butyricum* regulates lipid metabolism. In addition to its direct effects on lipid metabolism, *C. butyricum* has also been reported to prevent lipid accumulation through enterohepatic immunoregulation. For example, *C. butyricum* reduces inflammatory cytokine levels in the intestine, liver, and white adipose tissue (Zhou et al. 2017, Liao et al. 2023, Choi et al. 2023). However, it remains to be clarified whether these immunomodulatory effects are direct or represent an indirect consequence of improved lipid metabolism mediated by *C. butyricum*.

Gut microbiota‐derived EVs are increasingly recognized as critical mediators through which the microbiota influences host signaling pathways and cellular functions. Accumulating evidence indicates that these EVs play an essential role in maintaining intestinal homeostasis by protecting the epithelial barrier and enhancing immune responses, thereby contributing to the prevention of intestinal disorders such as colitis, diarrhea, and post‐infectious irritable bowel syndrome (Díaz‐Garrido et al. 2021, Melo‐Marques et al. 2024, Li et al. 2023, Zheng et al. 2023, Ma et al. 2022). The functional properties of EVs are largely determined by the composition of their cargo, which mainly includes nucleic acids, proteins, lipids, and peptidoglycans. Notably, PA encapsulated in *L. johnsonii*‐derived EVs was shown to alter CD36 palmitoylation and activate PPARγ signaling, thereby promoting lipid synthesis in mammary epithelial cells (Li et al. 2025), suggesting a potential role of EVs in lipid metabolism. In addition, RNAs carried by *L.murinus*‐derived EVs were found to target genes encoding enzymes involved in polyamine metabolism, and the resulting reduction in polyamine levels impaired recovery from dextran sodium sulfate‐induced colitis (Fan et al. 2024). These findings indicate that bacterial EV‐encapsulated RNAs may enter host cells and modulate the expression of target genes. We demonstrated that *C. butyricum*‐derived EVs are present throughout the intestine and can enter epithelial cells to regulate lipid metabolism. Notably, several milRNAs encapsulated within these EVs predominantly suppressed the expression of genes involved in the PPARα signaling pathway. Collectively, our results provide insights into how gut microbiota communicate with host cells to regulate lipid metabolism.

PPARα has been reported to play a critical role in intestinal lipid absorption, oxidation, and secretion. However, the effects of intestinal PPARα on lipid accumulation and obesity remain controversial. Activation of PPARα via specific agonists has been shown to reduce lipid accumulation and exert anti‐obesity effects (Hou et al. 2024, Araki et al. 2018). In contrast, intestinal PPARα ablation decreased systemic lipid absorption and improved hepatic steatosis (Stojanović et al. 2021, Yan et al. 2023). These discrepancies may result from functional overlap among the three PPAR isoforms, as most PPAR agonists and antagonists can target multiple isoforms simultaneously, whereas complete deletion of PPARα may trigger compensatory upregulation of PPARγ and PPARδ (Willson et al. 2000). In our study, milRNA derived from *C. butyricum* specifically targeted PPARα without affecting the other two isoforms, and our results further support the anti‐obesity role of PPARα through inhibition of lipid uptake in the small intestine.

It is noteworthy that we identified three *C. butyricum*‐derived milRNAs capable of modulating lipid metabolism. Among these, only milRNA‐3020179, which primarily targets *PPARα* and *Rxra*, was knocked out. We did not delete all three milRNAs because removing multiple fragments could potentially interfere with other physiological functions of *C. butyricum*. Nevertheless, the other two milRNAs, which target genes such as *Rxra*, *Rxrb*, and *Lpl*, did not appear to affect intestinal lipid transport. PPARα interacts with RXR to form a heterodimeric PPARα/RXR complex that subsequently regulates the transcription of downstream target genes (IJpenberg et al. 2004, Gervois et al. 2000). Given that there are three RXR isoforms (Rxra, Rxrb, and Rxrg), it is plausible that the inhibition of Rxra may be compensated by an upregulation of Rxrg expression. Consequently, suppression of Rxra and Rxrb, but not PPARα, did not markedly impact lipid metabolism. However, lipid metabolic alterations in peripheral tissues beyond the intestine were not assessed in the present study. Future investigations are therefore needed to determine whether *C. butyricum*‐derived EVs exert systemic effects on host lipid metabolism. In addition, the potential immunomodulatory mechanisms accompanying the observed metabolic improvements remain unclear and warrant further exploration. Finally, in vivo studies using intestine‐specific PPARα activation models will be required to further establish the role of the milRNA–PPARα axis in the regulation of lipid metabolism.

Furthermore, several limitations should be acknowledged. First, because the molecular mechanisms underlying the production and processing of miRNA‐like molecules in bacteria remain largely unclear, there is currently no standardized bacterial complementation system that can ensure the correct expression and maturation of such sequences. Consequently, genetic complementation was not performed to verify the specificity of the phenotypes observed in the knockout bacterial strains. Nevertheless, in the absence of rescue experiments, alternative explanations for the observed phenotypes cannot be completely excluded. In particular, deletion of the milRNA‐3020179‐containing genomic region may exert polar effects, alter local transcriptional regulation, or induce unintended secondary changes in bacterial physiology, which could subsequently influence EV biogenesis, composition, or function. Therefore, the current findings support an association between deletion of the investigated genomic region and the observed phenotypes, but do not yet provide definitive evidence for a causal role of milRNA‐3020179. Second, our study primarily focused on miRNAs, given that they are the most abundant and extensively characterized RNA species in EVs. Nevertheless, EVs harbor a diverse repertoire of RNA molecules, including piRNAs, tRNAs, snRNAs, Y RNAs, circRNAs, snoRNAs, lncRNAs, mRNAs, and rRNAs (Miceli et al. 2024). Although our functional assays indicated that RNAs within the 40–200 nt fraction did not influence lipid metabolism, other small RNAs (<40 nt, such as piRNAs and tRNA‐derived fragments) as well as longer RNAs (>200 nt, including circRNAs and lncRNAs) may still contribute to gene regulatory processes. Due to their relatively lower abundance, shorter half‐lives, and the limited functional evidence currently available in this context, systematically dissecting their roles was beyond the scope of the present study. Furthermore, although the observed biological activity was associated with the 20–40 nt RNA fraction, we cannot completely exclude the possibility that some small RNAs may originate from the degradation or processing of longer precursor RNAs. Consequently, additional studies are required to determine the origin of these small RNAs and to evaluate the potential regulatory functions of longer RNA species present in EVs. Third, in addition to EVs, microbiota‐derived metabolites are also known to influence host lipid metabolism and intestinal barrier function (Shelton et al. 2023, Liu et al. 2025). Although our findings provide substantial evidence that microbiota‐derived EVs play the critical role in regulating intestinal lipid absorption, the potential contribution of microbial metabolites cannot be completely excluded. Further studies are therefore required to dissect the relative contributions of EVs and microbial metabolites to host lipid metabolic regulation.

In conclusion, our study demonstrates that fat digestibility is closely linked to the composition of the gut microbiota. Notably, individuals with lower fat digestibility exhibited a higher abundance of *C. butyricum* in the ileal mucosa, and administration of *C. butyricum* suppressed intestinal lipid absorption and oxidation. Furthermore, small RNAs encapsulated within *C. butyricum*‐derived EVs represent candidate factors involved in the modulation of host intestinal lipid metabolism and the attenuation of lipid accumulation in both intestinal organoids and HFD‐fed mice. Collectively, these findings deepen our understanding of how gut microbes regulate host lipid metabolism and offer valuable insights for developing microbiota‐based strategies to combat obesity.

## Author Contributions

Hong Hu: conceptualization, methodology, data curation, software, investigation, writing – original draft. **Yiwen He**: conceptualization, investigation, writing – original draft, methodology, software, data curation. Jing Liang: data curation, formal analysis, investigation, methodology, software. Qi Han: methodology, software, data curation, formal analysis, investigation. **Xuqing Liang**: investigation, software, validation. **Guangtian Cao**: investigation, validation, software. **Yulong Tang**: methodology, visualization. **Liuqin He**: investigation, project administration, writing – review and editing, resources. **Yulong Yin**: conceptualization, funding acquisition, project administration, writing – review and editing. Xihong Zhou: conceptualization, funding acquisition, investigation, project administration, resources, writing – review and editing, supervision.

## Funding

This study was financially supported by National Key Research and Development Program of China (2022YFD1301500), National Natural Science Foundation of China (32341053), the Science and Technology Innovation Program of Hunan Province (2023RC1074), the Key Fundamental Research Program of Hunan Province (2024JC0007), and China Agriculture Research System of MOF and MARA (CARS‐35).

## Ethics Statement

Animal procedures were approved by the Protocol Management and Review Committee of the Institute of Subtropical Agriculture, Chinese Academy of Sciences. All animal experiments were approved under the general ethical approval number 20230126. The specific ethical approval numbers for individual animal experiments were 20230126‐1, 20230126‐2, 20230126‐3, 20230126‐4, 20230126‐5, 20230126‐6, and 20230126–7, respectively.

## Conflicts of Interest

The authors declare no Conflicts of Interest.

## Supporting information




**Supporting Information**: jev270381‐sup‐0001‐SuppMat.docx

## Data Availability

The raw data from 16S rRNA gene sequencing, transcriptome sequencing, and RNA sequencing have been deposited in the Genome Sequence Archive of the National Genomics Data Center, China National Center for Bioinformation (Beijing), under accession number CRA053815, CRA052414, and CRA052388, which are publicly accessible at https://ngdc.cncb.ac.cn/gsa. The remaining data that support the findings of this study are available within the article, supplementary information, or Source Data file. Source data are provided with this paper.
