# Supplementary material for: Extracellular Vesicle‐Encapsulated Small RNAs From Clostridium Butyricum Modulate Host Intestinal Lipid Metabolism
Source: J Extracell Vesicles. 2026 Sep 28;15(10):e70381. doi: 10.1002/jev2.70381 (PMC13619148; doi:10.1002/jev2.70381)
Supplement: Supplementary file 1 [file JEV2-15-e70381-s001.docx]

**Supplementary Table 1 Primer sequences for RT-qPCR analysis of gene expression**

| Gene | 5’-3’ Primer sequence | Gene | | 5’-3’ Primer sequence |
| --- | --- | --- | --- | --- |
| *CD36*, pig | F: tgacccagcacttgaagcaaa | | *Cd36*, mouse | F: gagccatctttgagccttca |
|  | R: ttccaagaggtatgcttcttttcca | |  | R: tcagatccgaacacagcgta |
| *FATP4*, pig | F: agctgcataaaacagggact | | *Fatp4*, mouse | F: gattcagagccctagcgtcc |
|  | R: atccagcatctgttggcctc | |  | R: ctgctagctttggtctggca |
| *FABP2*, pig | F: gcctggaagatagaccgcaa | | *Fabp2*, mouse | F: tgctgtccgagaggtttctg |
|  | R: tcatgagctgcaagtttcctct | |  | R: agaatcgcttggcctcaact |
| *FASN*, pig | F: cgttgggtcgactcactgaa | | *Fasn*, mouse | F: cccctcacatcaagtgggac |
|  | R: gagacagttcaccatgccca | |  | R: ttgggaagtcctcagcaacc |
| *SCD*, pig | F: gagtcaatgctcacggctga | | *Scd1*, mouse | F: agagaactggagacgggagtc |
|  | R: ttccctttcgagtgaggcac | |  | R: aaacaggaactcagaagcccaa |
| *CPT1A*, pig | F: tggtgtccaaatacctcgcc | | *Cpt1a*, mouse | F: gactccgctcgctcattcc |
|  | R: gataatcgccacggctcaga | |  | R: ttgagggcttcatggctcag |
| *PPARα*, pig | F: gaagtacggcgtctacgagg | | *PPARα*, mouse | F: aactgacgtttgtggctggt |
|  | R: cgcaccaaatgatagcagcc | |  | R: ctgtgatgacagagccctcg |
| *RXRa*, pig | F: tccttctcccaccgctccatc | | *Rxra*, mouse | F: tccttctcccaccgctccatc |
|  | R: cagctccgtcttgtccatctg | |  | R: cagctccgtcttgtccatctg |
| *LPL*, pig | F: tgccacgacaagtctctgaa | | *Lpl*, mouse | F: ttgcagagagaggactcgga |
|  | R: tagactccacgtgctgttcc | |  | R: agatgaatggagcgctcgtg |
| *PLIN1*, pig | F: tggtggcgtctgtatgcaat | | *Rxrb*, mouse | F: cagcggaatcgctgtcagta |
|  | R: agcaccgaggactttgtctg | |  | R: gagggaccgatcaaagatggc |
| *β-actin*, pig | F: tccctggagaagagctacga | | *PPARγ*, mouse | F: agatcatctacacgatgctggcct |
|  | R: tgttggcgtagaggtccttc | |  | R: tgtcctcgatgggcttcacgttca |
| *PPARδ*, mouse | F: cccgggaagaggagaaagag | | *β-actin*, mouse | F: tgtccaccttccagcagatgt |
|  | R: aaagcggatagcgttgtgc | |  | R: agctcagtaacagtccgcctaga |

**Supplementary Table 2 Primer sequences for *C. butyricum* quantification**

| Gene | 5’-3’ Primer sequence |
| --- | --- |
| *16S C. butyricum* | F: acctaaggaggaagccacgg |
|  | R: cgcccagtaaatccggacaa |
| *16S Universal* | F: cggcaacgagcgcaaccc |
|  | R: ccatgtagcacgtgtgtagcc |

**Supplementary Table 3 Sequences for identification of milRNA by RT-qPCR analysis**

|  | 5’-3’ Primer sequence |
| --- | --- |
| MilRNA-3047216 | F: aggcgtgcgaatatctggctg |
|  | R: atccagtgcagggtccgagg |
|  | RT: gtcgtatccagtgcagggtccgaggtattcgcactggatacgacgttata |
| MilRNA-3020179 | F: gcgcgaaggtctgagtatagc |
|  | R: atccagtgcagggtccgagg |
|  | RT: gtcgtatccagtgcagggtccgaggtattcgcactggatacgacgaaaaa |
| MilRNA-409778 | F: aagagcgttcgaacccctgatc |
|  | R: atccagtgcagggtccgagg |
|  | RT: gtcgtatccagtgcagggtccgaggtattcgcactggatacgacggtgcg |

**Supplementary Table 4 Sequences for milRNA agomir**

|  | 5’-3’ Primer sequence |
| --- | --- |
| MilRNA-3047216 | ucgaaccccugaucucgcacc  /i2OMeU/*/i2OMeG/*/i2OMeC//i2OMeG//i2OMeA//i2OMeG//i2OMeA//i2OMeU//i2OMeC//i2OMeA//i2OMeG//i2OMeG//i2OMeG//i2OMeG//i2OMeU//i2OMeU//i2OMeC//i2OMeG/*/i2OMeA/*/i2OMeU/*/i2OMeU/*(3`chol) |
| MilRNA-3020179 | aaggucugaguauagcuuuuuc  /i2OMeA/*/i2OMeA/*/i2OMeA//i2OMeA//i2OMeG//i2OMeC//i2OMeU//i2OMeA//i2OMeU//i2OMeA//i2OMeC//i2OMeU//i2OMeC//i2OMeA//i2OMeG//i2OMeA//i2OMeC//i2OMeC//i2OMeU/*/i2OMeU/*/i2OMeU/*/i2OMeU/*(3`chol) |
| MilRNA-409778 | ucgaaccccugaucucgcacc  /i2OMeU/*/i2OMeG/*/i2OMeC//i2OMeG//i2OMeA//i2OMeG//i2OMeA//i2OMeU//i2OMeC//i2OMeA//i2OMeG//i2OMeG//i2OMeG//i2OMeG//i2OMeU//i2OMeU//i2OMeC//i2OMeG/*/i2OMeA/*/i2OMeU/*/i2OMeU/*(3`chol) |
| Negative Control | uuguacuacacaaaaguacug |

**Supplementary Table 5 Primer sequences used for the quantification of genes adjacent to milRNA-3020179 in *C. butyricum***

| Gene | 5’-3’ Primer sequence |
| --- | --- |
| *aepX* | F: gcggtacccttctttcaagc |
|  | R: gccttattgcaaaggctggc |
| *EBQ27_17690* | F: atcatcaatttctccccacact |
|  | R: aatgccatttttgatgcggc |
| *EBQ27_17695* | F: tgtcgcttcccattttgcag |
|  | R: tggcaagtttagcagggtgt |
| *EBQ27_17700* | F: acccatgctcctgttgaatct |
|  | R: gcaatgcttacaggatggca |
| *EBQ27_17705* | F: tgctcccattcccataccag |
|  | R: gatgtgctgcagtgtaccca |
| *EBQ27_17710* | F: ctcaagacagcaacaacagca |
|  | R: tggcagatggggagtacgaa |
| *16S rRNA* | F: aatggaggaagccacttcgg |
|  | R: agccctagacataaggggca |

**Supplementary Figure 1 Representative images showing the results of density-based purification of Dil-labeled EVs**

Purified EVs were incubated with 5 μM DiI (Solarbio, Beijing, China) at 37 °C for 20 min in the dark. Following labeling, the samples were ultra-centrifuged at 150,000 *g* for 3 h at 4 °C to remove unbound dye. The resulting EV pellet was resuspended in 100 μL sterile PBS. To further eliminate residual free dye and dye aggregates, the labeled EVs were subjected to OptiPrep density gradient purification. A discontinuous iodixanol gradient consisting of 50%, 40%, 30%, 20%, and 10% (w/v) OptiPrep solutions was carefully prepared in ultracentrifuge tubes (Beckman Coulter) by layering the solutions sequentially from the bottom to the top. The 100 μL suspension of dye-labeled EVs was gently loaded onto the top of the gradient and centrifuged at 150,000 *g* for 3 h at 4 °C. After centrifugation, the EV-containing fraction was collected from the interface between the 20% and 30% iodixanol layers, whereas denser non-vesicular materials and dye aggregates remained in lower-density fractions, primarily below the 40% iodixanol layer, and were discarded. The collected EV fraction was diluted in 25 mL PBS and ultracentrifuged again at 150,000 *g* for 3 h at 4 °C to remove iodixanol and any residual free dye. Finally, the purified EV pellet was resuspended in 100 μL PBS and stored at −80 °C until further use.

**Supplementary Figure 2 Lipid digestion ability in piglets with different apparent crude fat digestibility**

(A) Ileum bile acids content; (B) Lipase activity; (C) Serum SCT content; (D) Serum CCK content; (E) Relative expression of genes involved in fat oxidation and synthesis; (F) Relative expression of genes involved in lipid transport. (Data were analyzed by two-tailed Student’s t-test, n = 10). Values are presented as mean ± SEM. ^*^*P* < 0.05. ^**^*P* < 0.01.

**Supplementary Figure 3 Alpha and beta diversity of gut microbiota across different intestinal regions**

(A) Shannon index; (B) Simpson index; (C) ACE index; (D) Chao1 index; (E) PCoA based on unweighted UniFrac distance. HFDM, Duodenal mucosa of piglets with higher apparent crude fat digestibility; LFDM, Duodenal mucosa of piglets with lower apparent crude fat digestibility; HFJM, Jejunal mucosa of piglets with higher apparent crude fat digestibility; LFJM, Jejunal mucosa of piglets with lower apparent crude fat digestibility; HFIM, Ileal mucosa of piglets with higher apparent crude fat digestibility; LFIM, Ileal mucosa of piglets with lower apparent crude fat digestibility; HFCM, Cecal mucosa of piglets with higher apparent crude fat digestibility; LFCM, Cecal mucosa of piglets with lower apparent crude fat digestibility; HFJC, Jejunal content of piglets with higher apparent crude fat digestibility; LFJC, Jejunal content of piglets with lower apparent crude fat digestibility; HFIC, Ileal content of piglets with higher apparent crude fat digestibility; LFIC, Ileal content of piglets with lower apparent crude fat digestibility; HFCC, Cecal content of piglets with higher apparent crude fat digestibility; LFCC, Cecal content of piglets with lower apparent crude fat digestibility; HFCoC, Colonic content of piglets with higher apparent crude fat digestibility; LFCoC, Colonic content of piglets with lower apparent crude fat digestibility.

**Supplementary Figure 4 Gut microbial abundance across different intestinal regions**

(A) The relative abundance of gut microbiota at the phylum level; (B) The relative abundance of gut microbiota at the genus level.

**Supplementary Figure 5 Lipid content in the liver of recipient mice**

(A) Hepatic TG content; (B) Hepatic NEFA content. TG, triglycerides; NEFA, non-esterified fatty acid. LOW, Piglets with lower apparent crude fat digestibility; HIGH, Piglets with higher apparent crude fat digestibility; LFJM, Mice colonized with jejunal mucosal microbiota from LOW piglets; HFJM, Mice colonized with jejunal mucosal microbiota from HIGH piglets; LFIM, Mice colonized with ileal mucosal microbiota from LOW piglets; HFIM, Mice colonized with ileal mucosal microbiota from HIGH piglets. (Data were analyzed by one-way ANOVA, n = 8). Values are presented as mean ± SEM.

**Supplementary Figure 6 Factors associated with lipid digestion in *C. butyricum*-treated mice**

(A) Fecal bile acid content; (B) Fecal lipase activity; (C) Serum SCT content; (D) Serum CCK content. SCT, Secretin; CCK, Cholecystokinin. (Data were analyzed by two-tailed Student’s t-test, n = 8). Values are presented as mean ± SEM.

**Supplementary Figure 7 Sodium butyrate did not affect intestinal lipid metabolism**

(A) Serum TG content; (B) Serum NEFA content; (C) Serum TC content; (D) Fecal TG content; (E) Fecal NEFA content; (F) Fecal TC content; (G) Relative expression of genes involved in lipid transport; (H) Relative expression of genes involved in fat oxidation and synthesis. CONT, control mice; BUTY, mice treated with sodium butyrate. (Data were analyzed by two-tailed Student’s t-test, n = 6). Values are presented as mean ± SEM.

**Supplementary Figure 8 Orally administered EVs detected in the intestine**

DiR-labeled EVs administered orally to mice were detectable in the intestine at 6 h and were largely excreted by 24 h, whereas free DiR was completely excreted within 6 h after oral administration.

(A) Negative control; Orally administered DiR-labeled EVs after 3 h (B), 6 h (C) and 24 h (D); Orally administered free DiR dye after 3 h (E) and 6 h (F). Red and blue, EVs.

**Supplementary Figure 9 Cell viability assay of EV treatment in IPEC-J2 cells**

IPEC-J2 cells were treated with different concentrations of EVs for 24 h (A) and 48 h (B). Data were analyzed by one-way ANOVA (n = 6). Values are presented as mean ± SEM.

**Supplementary Figure 10 Original representative images of Oil red O staining results.**

CONT, control cells; EVS, cells treated with EVs; Wy14643, cells treated with both EVs and the PPARα agonist Wy14643. scale bar = 15 μm.

**Supplementary Figure 11 Expression of** **milRNA-3020179 in EVs treated with RNase**

(A) Ct values of RT–qPCR analysis of milRNA-3020179 in EVs following different treatments. (B) Agarose gel electrophoresis of RT–qPCR products for milRNA-3020179 following different treatments.

RNase, EVs treated with RNase; Triton, EVs treated with Triton; Rnase and Triton, EVs treated with RNase and Triton; Buffer, EVs treated with buffer and Triton. Data were analyzed by one-way ANOVA (n = 3). Values are presented as mean ± SEM.

**Supplementary Figure 12 Effects of EV pre-treatment on lipid metabolism in cells**

(A) TG content in IPEC-J2 cells; (B) TG content in the culture medium; Relative expression of *LPL* (C), *CD36* (D), *FATP4* (E), *FABP2* (F), *FASN* (G), *CPT1A* (H), *SCD* (I), *PPARα* (J) and *RXRa* (K) genes; (L) FAO activity. (Data were analyzed by one-way ANOVA, n = 4). Values are presented as mean ± SEM. ^*^*P* < 0.05. ^**^*P* < 0.01. ^***^*P* < 0.001.

**Supplementary Figure 13 Effects of EV RNA fractions (20–40 nt and 40–200 nt) on lipid metabolism in cells**

(A) TG content in IPEC-J2 cells; (B) TG content in the culture medium; Relative expression of *LPL* (C), *CD36* (D), *FATP4* (E), *FABP2* (F), *FASN* (G), *CPT1A* (H), *SCD* (I), *PPARα* (J) and *RXRa* (K) genes; (L) FAO activity. (Data were analyzed by one-way ANOVA, n = 4). Values are presented as mean ± SEM. ^*^*P* < 0.05. ^**^*P* < 0.01. ^***^*P* < 0.001.

Small RNA fractions corresponding to 20–40 nt and 40–200 nt RNA fragments were separated by denaturing polyacrylamide gel electrophoresis. The RNA fractions were quantified using a NanoDrop spectrophotometer, and equal amounts of RNA (100 ng per well) from each fraction were transfected into IPEC-J2 cells using Lipofectamine RNAiMAX (Thermo Fisher Scientific) according to the manufacturer’s instructions. Briefly, IPEC-J2 cells were seeded in 24-well plates and cultured to approximately 70–80% confluence prior to transfection. For each well, RNA samples were diluted in Opti-MEM medium and gently mixed with Lipofectamine RNAiMAX reagent to allow formation of RNA–lipid complexes, which were incubated for 10–15 min at room temperature before being added dropwise to the cells. After transfection, cells were incubated under standard culture conditions (37 °C, 5% CO₂) for 24 h unless otherwise specified, after which downstream analyses, including assessment of fatty acid oxidation activity and lipid metabolism-related gene expression, were performed. All experimental groups were treated under identical conditions to ensure comparability, with equal RNA input used for each fraction to exclude potential bias caused by dosage differences.

**Supplementary Figure 14 Length distribution of small RNAs mapped to the *C. butyricum* reference genome**

**Supplementary Figure 15 Original representative images of IPEC-J2 cells incubated with Cy5-labeled milRNA-3020179**

Representative fluorescence images of IPEC-J2 cells incubated with Cy5-labeled milRNA-3020179 for 6 h. Cells treated with transfection reagent alone under identical conditions served as the negative control. After incubation, cells were washed, fixed, and stained with DAPI. Red fluorescence indicates Cy5-labeled milRNA-3020179, and blue fluorescence indicates nuclei. Transfection reagent, cells treated with transfection reagent alone; Cy5-labeled milRNA, cells treated with transfection reagent and Cy5-labeled milRNA-3020179. Scale bar = 5 μm.

**Supplementary Figure 16 Detection of milRNAs in the ileum after oral administration of agomirs**

Agomir, mice received a mixture of three milRNA agomirs (milRNA-409778, milRNA-3020179, and milRNA-3047216), with each agomir administered at 20 mg/kg body weight (total dose: 60 mg/kg body weight); NC, mice received negative control at the same total dose (60 mg/kg body weight); PBS, mice received an equal volume of PBS. At the end of the experiment, all animals were sacrificed, and ileal samples were immediately collected for RNA extraction. Total RNA was then used for miRNA cDNA synthesis using the TaqMan Advanced miRNA cDNA Synthesis Kit (Applied Biosystems). Quantitative PCR was performed with the TaqMan Advanced miRNA Assay Kit (Applied Biosystems) on a Roche LightCycler 480 II system according to the manufacturer’s instructions. Primer sequences are listed in Supplementary Table 3. PCR products were subsequently analyzed by agarose gel electrophoresis.

**Supplementary Figure 17** **Sequencing of the target sequence encoding milRNA-3020179 in C. butyricum**

WT, wild-type C. butyricum; Gene-editing, milRNA-3020179 knockout strain of *C. butyricum*.

**Supplementary Figure 18 Detection of milRNA-3020179 in EVs derived from C. butyricum**

EVS, EVs derived from wild-type C. butyricum; EVS-KO, EVs derived from milRNA-3020179 knockout strain of *C. butyricum*.

**Supplementary Figure 19 Growth curve and relative expression levels of three upstream and three downstream genes flanking milRNA-3020179 in the KO *C. butyricum* strain**

(A) Growth curve; Relative gene expression of *aepX* (B), *EBQ27_17690* (C), *EBQ27_17695* (D), *EBQ27_17700* (E), *EBQ27_17705* (F), and *EBQ27_17710* (G). Values are presented as mean ± SEM. n = 4.
